# Development of a standardized role script template for simulated participant scenarios – results of a multi-step consensus process in the German-speaking countries

**DOI:** 10.3205/zma001812

**Published:** 2026-02-17

**Authors:** Tim Peters, Daniel Bauer, Angelika Hiroko Fritz, Sandra Hahn, Linn Hempel, Loretta Reck, Miriam Reicherts, Andrea Schönbauer, Renate Strohmer, Christian Thrien, Michael Weber, Anja Zimmermann, Elvira Pippel

**Affiliations:** 1 Bielefeld University, Medical School OWL, Department of Studies and Teaching, Department of General Practice and Family Medicine, Bielefeld, Germany; 2 University of Bern, Faculty of Medicine, Institute for Medical Education, Bern, Switzerland; 3 University of Duisburg-Essen, University Hospital Essen, Simulation Person Program, Faculty of Medizin, Essen, Germany; 4 German Institute for State Examinations in Medicine, Pharmacy, Dentistry and Psychotherapy (IMPP), Mainz, Germany; 5 University of Halle-Wittenberg, Medical Faculty, Dorothea Erxleben Learning Center Halle, Halle (Saale), Germany; 6 Mutterstadt, Germany; 7 University of Augsburg, Faculty of Medicine, Department of Medical Education (DEMEDA), Augsburg, Germany; 8 Philipps-University Marburg, Dr. Reinfried Pohl-Center of Medical Education, Marburg, Germany; 9 Medical Faculty Mannheim of the Heidelberg University, Division of Studies and Teaching Development, SkillsLab TheSiMa, Simulation Person Program, Mannheim, Germany; 10 University of Cologne, Faculty of Medicine, Cologne Interprofessional Skills Lab and Simulation Centre, Cologne, Germany; 11 University of Bonn, Faculty of Medicine, Dean’s Office, Simulation Person Program, Bonn, Germany; 12 Charité – Universitätsmedizin Berlin, corporate member of Freie Universität Berlin, Humboldt-Universität zu Berlin, Office for Study Affairs, Berlin, Germany; 13 Uppsala County, Sweden

**Keywords:** simulated participants, human simulation, interprofessional education, health profession education

## Abstract

**Aim:**

The need for a scientifically founded role script template for simulated participants (SPs) arises from the great importance that SPs have for teaching and assessments in the health professions. Stakeholders have thus far developed their own scripts, making usage across institutions and professions difficult. The aim of this research project is therefore to develop an evidence-based, interprofessional role script template for simulated participants.

**Method:**

To integrate the diverse traditions and needs of the professional groups, a multi-stage developmental and consensus process, based on the Delphi method, was conducted by a working group of 19 members over 10 rounds. This process incorporated, among other inputs, the findings of a systematic literature review, feedback from a 24-member interprofessional expert panel, and 11 reviews by experienced SPs.

**Results:**

The template has 13 categories, and its modular structure allows for flexible use in teaching and assessment, vocational training, academic studies, as well as further education and training. The template is designed to be consistently interprofessional and suitable for use in the fields of occupational therapy, midwifery, medicine, speech therapy, nursing science, pharmacy, physiotherapy, psychotherapy, emergency response services, veterinary medicine, and dentistry.

**Conclusion:**

The complex consensus process reflected the heterogeneity of SP practice in the health professions. Nevertheless, experiences were compiled in one template so that it is possible to interchange scripts between the professions and across institutions and to (further) develop them collectively. The next steps are to evaluate the template's usability and its ongoing development in the community of practice.

## Zusammenfassung

**Zielsetzung:**

Aus der großen Bedeutung von Simulationspersonen (SP) für Lehre und Prüfungen in den Gesundheitsberufen ergibt sich die Notwendigkeit eines wissenschaftlich fundierten Templates für SP-Rollenskripte. Bisher entwickeln Akteure eigene Vorlagen, was eine standort- und berufsgruppenübergreifende Nutzung erschwert. Ziel des Forschungsprojektes ist es daher, ein evidenzbasiertes, interprofessionelles Template für Rollenskripte zu entwickeln.

**Methodik:**

Um die heterogenen Traditionen und Bedarfe der Berufsgruppen zusammenzuführen, wurde in einer 19 Personen umfassenden Arbeitsgruppe ein an die Delphi-Methode angelehnter, mehrstufiger Erarbeitungs- und Konsensprozess über 10 Runden durchgeführt. Einbezogen wurden u. a. die Ergebnisse einer systematischen Literaturrecherche, Rückmeldungen eines 24-köpfigen interprofessionellen Expert:innen-Panels sowie 11 Reviews erfahrener SP.

**Ergebnisse:**

Das Template enthält 13 Kategorien und ein modularer Aufbau erlaubt eine flexible Nutzung in Lehre und Prüfung, Ausbildung und Studium sowie in der Fort- und Weiterbildung. Das Template ist konsequent interprofessionell gestaltet und kann in den Fachbereichen Ergotherapie, Hebammenwissenschaft, Humanmedizin, Logopädie, Pflegewissenschaft, Pharmazie, Physiotherapie, Psychotherapie, Rettungswesen, Veterinärmedizin und Zahnmedizin genutzt werden.

**Schlussfolgerung:**

Der aufwändige Konsensprozess zeigte die Heterogenität von SP-Einsätzen in den Gesundheitsberufen. Gleichzeitig konnten Erfahrungen in einem Template zusammengeführt werden, dass es erlaubt, Rollenskripte professions- und standortübergreifend auszutauschen und gemeinsam (weiter) zu entwickeln. Die nächsten Schritte sind eine Evaluation der Usability und eine Weiterentwicklung in der community of practice.

## 1. Introduction

Working with simulated participants (SPs) is widely established internationally in healthcare education [1]. Written role scripts are used to develop and define case content and pedagogical objectives of SP-based scenarios, to train SPs, and to prepare them for the actual simulation encounters [2]. However, there are two challenges when developing and implementing these scripts.

First, in the context of teaching, but particularly in (high-stakes) examinations, internal and cross-station standardization plays a crucial role [3]. To achieve a good balance between standardization and role credibility in simulations, a template must contain relevant details about the role to be portrayed and the organizational context. Due to the lack of scientifically developed guidance, SP programs and other parties who work with SPs are left to their own resources. They each draft, usually for the first time, their own templates, often based on “good practice” versions from other institutions. As a consequence, practices unsupported by evidence are perpetuated and existing mistakes are repeated or copied. Moreover, modifications made to such templates during site-specific (further) development lead to a “backwards adjustment” of all of these scripts, which is a frequent source of error.

Second, difficulties arise when scripts are intended for cross-institutional or cross-professional use, as well as when they are to be further developed collectively by the community of practice [4]. Due to differing traditions and organizational structures, the ways in which SP are employed vary widely not just around the globe but also within German-speaking regions [5], [6]. Looking at SP scripts from different health professions, the scoping review by Davies et al. [7] shows that different curricula and teaching/learning structures encourage diversity among the scripts. Over the years, each institution and each profession has established its own template terminology and structure. This makes it difficult to collaborate across professions and disciplines when developing SP role scripts, and thus also hampers SP activities based on them.

In addition to frequently unanswered questions regarding authorship or sharing scripts with third parties, the diversity mentioned above and the monoprofessional focus of templates result in SP role scripts that can only very rarely be interchanged and used across institutions. Interprofessional use of role scripts generally does not occur in cross-site teaching or assessment formats. Moreover, the frequent lack of exchange means that external feedback for the continuous development and quality assurance of role scripts is missing.

The existing literature on role scripts reveals considerable heterogeneity. There are publications on SP role scripts [8], [9], [10], [11], general quality standards [5], [12] and concrete instructions and templates [13], [14]. These provide valuable guidance but, as they are usually tailored to English-speaking settings, can be applied to German-speaking countries only to a limited extent. The publications also frequently have a monoprofessional focus, are experience-based, and address, in part, specific individual aspects. There are also national and international training programs specialized in working with SPs, writing role scripts, and qualifying educators [15], [16], [17]. These make an important contribution to the professionalization of the work done with SPs, but in terms of scripted SP scenarios they are not yet thoroughly founded scientifically so that transferring them to complex simulations or high-stakes OSCEs (Objective Structured Clinical Examination) remains a challenge that still must be solved within the individual institutions.

The research team has therefore sought to develop an evidence-based template for SP role scripts. The questions to be answered were whether an interprofessional design is possible and how it would need to be structured in terms of form and content in order to meet the challenges identified above.

## 2. Method

To take the various health professions and the involved parties into account, a development and consensus process, based on the Delphi method [18], was conducted with 19 members of the DACH Association for Medical Education’s (GMA) Committee on Simulated Persons. The experiences and perspectives of external experts were also integrated into the process [19]. The following five steps were carried out consecutively, and the interim results were brought together in the interprofessional and international working group and consented upon over a total of 10 rounds.

### 2.1. Kick-off workshops

The initiative to critically address SP role scripts was spearheaded by the Institut für medizinische und pharmazeutische Prüfungsfragen (IMPP [German Institute for State Examinations in Medicine, Pharmacy, Dentistry and Psychotherapy]) and initially focused on the requirements that a template would have to meet for national licensing exams and the associated need for standardization. This phase benefited from the extensive experience of Swiss colleagues who have already employed SPs in the Swiss Federal Licensing Exam in Medicine for many years [20]. Prior to incorporating the literature review, three full-day workshops were conducted to identify relevant categories, cross-site standards and best-practice aspects for the German-speaking regions in an exploratory, inductive process and to generate a template as a prototype. 

### 2.2. Literature review

In a second step, a systematic literature review was carried out in the PubMed, CINAHL, PsycINFO and ERIC databases using common search terms and synonyms. Inclusion criteria were publications in German and English addressing the topic of human simulation. All types of articles, regardless of study design that had been published between January 1, 2003 and July 15, 2023 were taken into consideration. In addition, supplementary research of the internet was undertaken, and grey literature and handbooks with thematic relevance were viewed. The database searches did not yield any relevant results in regard to concrete role script templates for SPs. These could only be found in the published recommendations of relevant professional organizations [13], [14] and in handbooks [2], [10]. A total of 19 papers and 12 other grey literature publications were identified as useful for designing a SP role script template or that could be used as exemplary templates. These were considered when revising the template and discussed by the working group. Main sources are listed in the bibliography below and, if directly cited, in the template itself. Due to their thematic complexity, two topics (diversity in the SP pool and non-diagnostic interventions) were prepared for the template by sub-working groups.

### 2.3. Interprofessional expert review

To ensure the template could be used interprofessionally and interdisciplinarily, the template underwent a process of interprofessional review. At least two representatives for each field – occupational therapy, midwifery, medicine, speech therapy, nursing science, pharmacy, physiotherapy, emergency response services, veterinary medicine, and dental medicine – with experience employing SPs in teaching and assessment submitted a review. These 22 reviews were incorporated into the template.

### 2.4. SP review

Following this, a total of 11 experienced SPs from Germany and Switzerland reviewed the template. The SPs were between the ages of 28 and 67. The mean (MD) was 48 years; the standard deviation (SD) is 13.5 years. Central to this feedback were the level of manageability for the target group and gathering experience-based recommendations.

### 2.5. Peer review & piloting

The template was presented at a total of three international conferences (International Skills Lab Symposium in 2022 [21], the annual conference of the DACH Association for Medical Education in 2023 [22], and the annual conference of the Association for SP Educators in 2024 [23]). The template was also piloted at several institutions, including the third-party-funded project eKommMed.nrw [https://www.ekommmednrw.uni-bonn.de/]. The aim was to collect feedback regarding the template's applicability and usability in everyday practice.

**Figure 1:**
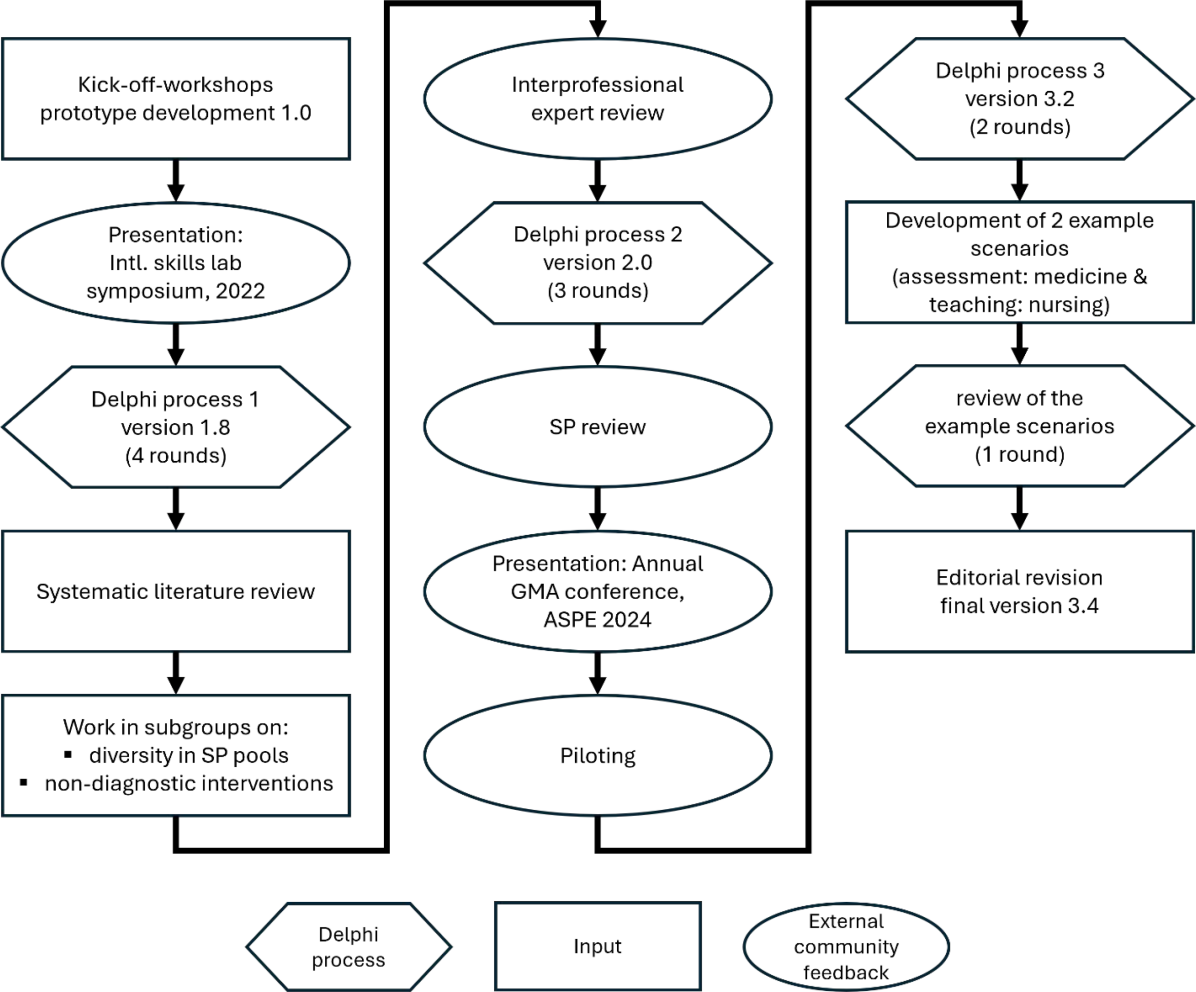
Flowchart of the development process

An overview of this process is presented in the flowchart (see figure 1 (Fig. 1)). For clarity, the work steps are divided according to the categories *inputs* (content and research originating from the working group), *Delphi process*, and *community feedback* (external feedback).

## 3. Results

When bringing the inputs, community feedback and feedback from the Delphi rounds together, the differences and main points of focus became clear. Recommendations in the literature usually reflect the perspective of SP programs or (medical) institutions. They focus, as also noted by Davies et al. [7], on demographic data, case histories, the correct presentation of symptoms, and organizational aspects [13]. Other main points focus on integrating the reason for the medical consultation with the role of the fictional character [2], anchoring the scenario in curricula and assessment [9], and the process of case scenario development [8], [10].

During the interprofessional expert review, technical terms and terminology specific to individual professions were flagged. Many health professions emphasize physiological progressions and non-disease-related reasons for consultations or appointments. Furthermore, the template categories were supplemented and expanded to include specific details and examples, e.g., regarding settings, specialized equipment, documented findings, accompanying persons or specific case histories. Physical examinations and diagnostic procedures differ depending on the established practices of a profession and its traditions regarding simulation which leads to adaptations and adjustments. The section concerning “diversity in the SP pool” was likewise expanded to include pregnant persons and people with communication difficulties.

In the SP reviews, the focus was placed on comprehensibility, consistency, and clarity. Also important was the transparency of the learning/assessment objectives and the organizational procedures. Lastly, aspects about being true to everyday life and structuring the template to fit the process of adopting a role and training for it were frequently commented on.

During the Delphi process itself the different practices and conventions between institutions became apparent in the working group. Elements such as complementary SP learning objectives or the nature of the simulated participant's motivation to speak were integrated into the template as a result. Experiences with certain topics (e.g., cultural competence, gender medicine) or specialties (e.g., psychiatry) also led to adaptations of the SP template.

The result of the process is a role script template for SPs in German and English that can be used for teaching and examinations. The full template and two example case scenarios for implementation (assessment: medicine; teaching: nursing) can be found in the supplementary material (see attachments 1 , attachment 2 and attachment 3 ).

**Figure 2:**
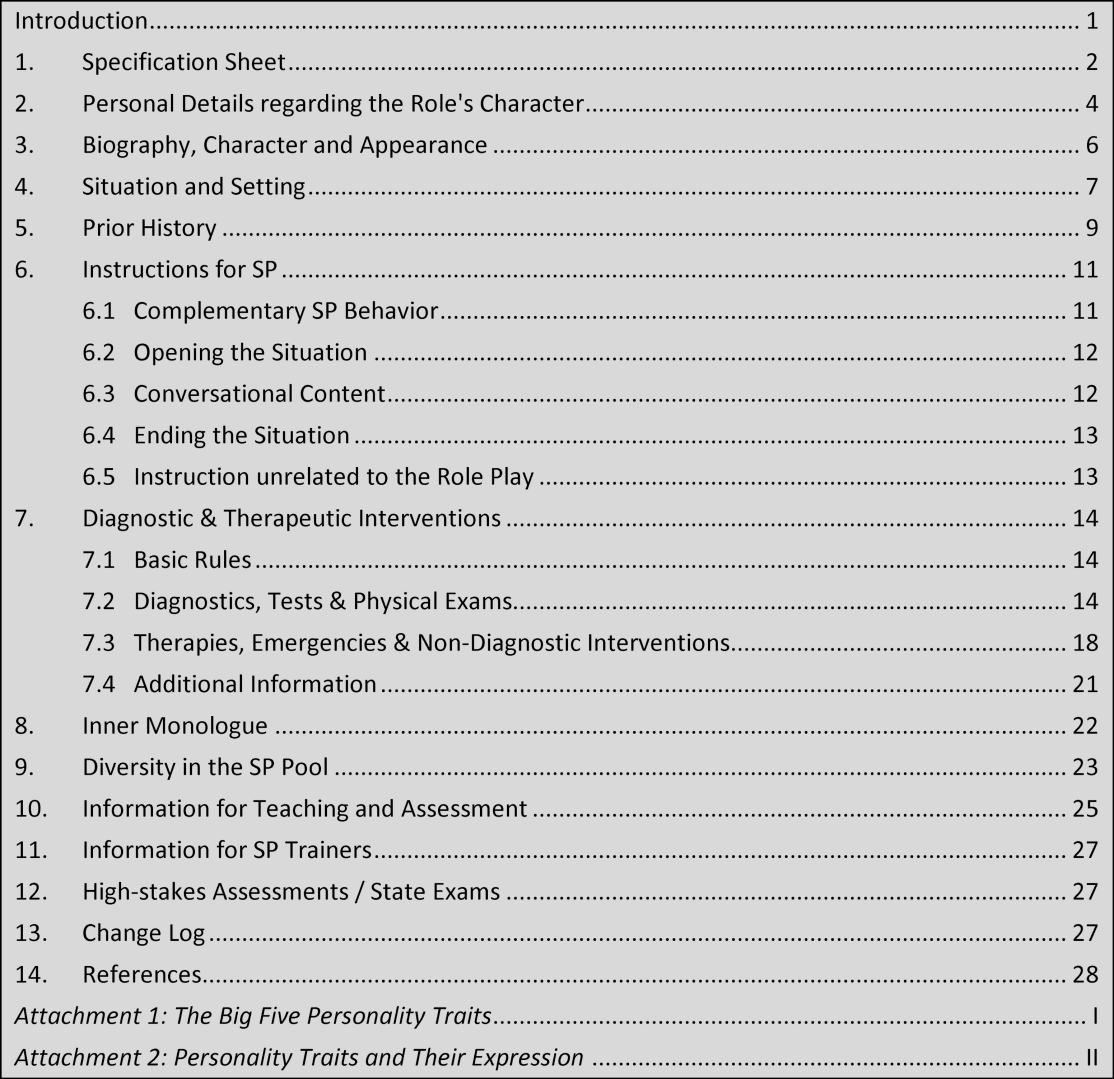
Template categories for SP role scripts

The template is comprised of 13 categories in total and has been given a modular structure to ensure that it remains manageable and can be tailored to the complexity of any given simulation. The template is consistently interprofessional in its structure and terminology and can be used in occupational therapy, midwifery, medicine, speech therapy, nursing science, pharmacy, physiotherapy, psychotherapy, emergency response services, veterinary medicine, and dentistry. Moreover, it can be used in vocational training, academic studies, and further education and training. In principle, its use would also be possible beyond health-specific contexts (e.g., social work, teacher training), though this was neither the focus of nor the intent behind the template’s development. The SP template includes the following categories (see figure 2 (Fig. 2)). 

## 4. Discussion

The aim of this research project was to develop a scientifically based, interprofessional role script template for SPs through a multi-stage development and consensus process based on the Delphi method. To this end, experienced experts from different professions and disciplines and SPs of diverse ages and backgrounds were recruited to participate. Taking the literature into consideration, the template presented here reflects the current agreement in the working group and the reviewers. It is provided as an Open Educational Resource (OER) to SP programs and enables the application of case scenarios across professions and institutions, which is also intended as a response to the call for interprofessional formats in teaching and assessment. Moreover, widespread use of the template facilitates simplified data collection for research projects.

The template aims to be as comprehensive as possible, but it may not include every element required for every conceivable SP assignment. Even so, it is not always necessary to complete the entire template. Hence, the template has been licensed under CC BY 4.0 [https://creativecommons.org/licenses/by/4.0/deed.en] and will be subject to ongoing development. It may be used, in part or in full, by others in their own work as it is, further developed, modified and disseminated, with proper citation of the original source.

During the drafting and development stage, it was not always possible to reach unanimous agreement. At points, there was contradictory feedback in the interprofessional expert reviews or in the Delphi rounds. Therefore, several decisions were made by the authors. In particular, these include:

### 4.1. Transparency of the learning objectives

The authors consider transparency of the learning and assessment objectives essential, as it helps SPs understand their roles in the simulations and incorporate this understanding into their performance [24]. It is also easier to avoid disparities and errors in portrayal, especially across multiple repetitions [25]. Besides, transparency is desired by the SPs.

### 4.2. Complementary SP behavior

Based on the student's learning objectives, complementary learning objectives for the SPs are defined to match [26], so that the often heterogeneous, SP-oriented training can be more clearly standardized, specified for and aligned with the simulations.

### 4.3. Representing diversity

The inclusion of diversity characteristics in SP case scenarios is explicitly supported by the current literature [27], [28]. Accordingly, various categories have been added to the template, accompanied by examples to facilitate their integration. Given the ongoing political and academic discussions surrounding diversity, the categories in the template are presented as nonbinding suggestions that may be adapted over time in response to evolving social developments.

### 4.4. Classification of personality traits

The elaboration of a persona's personality traits is handled very differently in practice. To allow for comparison here, reference is made in the template to the well-known OCEAN model [29], [30], [31] and its application is recommended.

### 4.5. Specific groups of SPs

Many publications address working with specific groups of SPs, e.g., seniors, children and adolescents, or people with cognitive impairments [32], [33], [34], [35]. The relevant aspects that should be documented in a role script have been included in the template. Organizational and training-related aspects were not included.

### 4.6. Safety precautions for SPs

Safety precautions for SPs have been recommended for a long time and are viewed as central to good collaboration [5], yet these are extremely heterogeneous and often insufficiently implemented [6]. Safety precautions are therefore mentioned as a main component in the template and their use is pointed out.

### 4.7. Inner monologue as an acting technique

Various acting techniques are applied by SPs and in role training [36]. These techniques require certain competencies on the part of SPs and/or SP trainers. The inner monologue has been prominently integrated into the template as an easily accessible and text-based technique because it can be learned easily and also used by amateur actors.

## 5. Conclusions

The development process shows that the heterogeneity of SP programs in regard to institutions and procedures in the various health professions influences the structure and content of the role scripts and thus confirms the findings of the scoping review by Davies et al. [7].

Nevertheless, it became clear that it is possible to create and use an evidence-based SP role template that encompasses multiple health professions and diverse levels of training and higher education. A consequence of broad usability, however, is the template’s considerable scope – a challenge that could be mitigated through modularization.

The template's strengths are that it takes relevant publications into consideration and is based on the experiences of different professions and SPs, all of which was compiled in a scientific manner. Also, the consistently interprofessional focus is an international novelty. The template thus opens opportunities to give different professions and institutions a common methodological base and to facilitate collaboration between programs of health professions education. The fact that the template is also available in German will encourage acceptance in the German-speaking countries. Furthermore, the template enables quality assurance and continuing professionalization of the work with SPs. As this is done, it can be helpful to define concrete quality requirements for the work performed by SP trainers and SPs and to design future advanced training and education on working with SPs.

The limitations needing to be pointed out include the wide scope, which can seem daunting at first, and several redundancies which, despite many revisions, could not be fully eliminated. Several of the contradictory reviews demonstrated that consensus was not always possible to reach. And several current topics, such as digital SP deployment and guidelines on anti-racist healthcare, are not yet sufficiently implemented and will require future revisions. Finally, at this point in time, despite the piloting a broad, systematic evaluation has not yet been conducted.

Given this latter issue, the next step will therefore be the broadest dissemination possible in Germany, Austria and Switzerland and an evaluation of the template's usability. Following this, the plan is to convert the template into a digital and, if necessary, database-supported version with plans for ongoing further development in the German-speaking community of practice.

## Acknowledgements

For their participation in the working group of the DACH Association for Medical Education’s (GMA) Committee on Simulated Persons (ASP), we thank Susanne Borgmann (Göttingen), Julia Freytag (Berlin), Regina Gramer (Tübingen), Daniela Mauer (Bonn), Florian Neubauer (Bern) and Stefanie Otten-Marré (Düsseldorf). We also thank the experts who reviewed the template: Occupational therapy: Annette Schüller (Bochum), Tina Stibane (Marburg); Midwifery: Nicola Bauer (Köln), Barbara Beck (Bochum), Ruth Berghoff (Bochum); Medicine: Robert Kleinert (Bielefeld), Barbara Woestmann (Bochum); Speech therapy: Juliane Leineweber (Göttingen), Corinna Fohler (Düsseldorf); Nursing science: Meike Schwermann (Münster), Claudia Schlegel (Bern); Pharmacy: Christoph Ritter (Greifswald), Sandra Wüst (Bern); Physiotherapy: Tim Herzig (Bielefeld), Marietta Handgraaf (Bochum); Psychology/Psychotherapy: Margarete Boos (Göttingen), Miriam Kunz (Augsburg), Carolyn Nelles (Brandenburg); Emergency response services: Kevin Stiller (Mannheim), Michael Langner (Wuppertal); Veterinary medicine: Simone Forterre (Bern), Christin Kleinsorgen (Hannover); Dentistry: Stefan Rüttermann (Frankfurt), Sabine Senhenn-Kirchner (Göttingen). And lastly, we thank the SPs for their reviews: Anja Krüger (Essen), Marcel Schäfer (Bochum), Reinhard Philipp (Mannheim), Bernd Wasser (Göttingen), Angelika Albrecht-Schaffer (Augsburg), Thomas Wißmann (Bonn), Finn Nachfolger (Bern), Rosanna Steyer (Berlin), Charlotte Welling (Düsseldorf), Lena Heikenfeld (Marburg), Christian Cujovic (Köln).

## Authors’ ORCIDs

- Tim Peters: [0009-0008-4165-5608]
- Daniel Bauer: [0000-0002-3337-3327]
- Linn Hempel: [0009-0009-5421-2029]
- Miriam Reicherts: [0009-0005-8867-6084]

## Competing interests

The authors declare that they have no competing interests. 

## Supplementary Material

Role script template for Simulated Participants (SPs)

Role script template for Simulated Participants (SPs). Example for an assessment in undergraduate medicine

Role script template for Simulated Participants (SPs). Example for teaching in nursing
