# Supplementary material for: Development of a standardized role script template for simulated participant scenarios – results of a multi-step consensus process in the German-speaking countries
Source: GMS J Med Educ. 2026 Feb 17;43(2):Doc18. doi: 10.3205/zma001812 (PMC12936913; doi:10.3205/zma001812)
Supplement: Role script template for Simulated Participants (SPs) [file JME-43-18-s-001.pdf]

# Attachment 1: Role script template for Simulated Participants (SPs)

Attachment 1 to Peters T, Bauer D, Fritz AH, Hahn S, Hempel L, Reck L, Reicherts M, Schönbauer A, Strohmer R, Thrien C, Weber M, Zimmermann A, Pippel E. *Development of a standardized role script template for simulated participant scenarios – Results of a multi-step consensus process in the German-speaking countries.* GMS J Med Educ. 2026;43(2):Doc18. DOI: 10.3205/zma001812

# Role script template

# for Simulated Participants (SPs)

## Version 3.4

dated 20 December 2024

# Table of Contents

Introduction ... 1
1. Specification Sheet ... 2
2. Personal Details regarding the Role's Character ... 4
3. Biography, Character and Appearance ... 6
4. Situation and Setting ... 7
5. Prior History ... 9
6. Instructions for SP ... 11
   6.1 Complementary SP Behavior ... 11
   6.2 Opening the Situation ... 12
   6.3 Conversational Content ... 12
   6.4 Ending the Situation ... 13
   6.5 Instructions unrelated to the Role Play ... 13
7. Diagnostic & Therapeutic Interventions ... 14
   7.1 Basic Rules ... 14
   7.2 Diagnostics, Tests & Physical Exams ... 14
   7.3 Therapies, Emergencies & Non-diagnostic Interventions ... 18
   7.4 Additional Information ... 21
8. Inner Monologue ... 22
9. Diversity in the SP Pool ... 23
10. Information for Teaching and Assessment ... 25
11. Information for SP Trainers ... 27
12. High-stakes Assessments / State Exams ... 27
13. Change Log ... 27
14. References ... 28
*Attachment 1: The Big Five Personality Traits* ... I
*Attachment 2: Personality Traits and Their Expression* ... II

# Introduction

This template is meant to (further) professionalize the development and quality assurance of role scripts for simulated participants (SPs) in health-related teaching and assessment, and to standardize them to enable interchangeability across disciplines and institutions.

As defined by the Open Educational Resources (OER), this template is offered to the different German-speaking simulated participant programs by the Committee on Simulated Persons (ASP) of the DACH Association for Medical Education (GMA) and the German Institute for State Examinations in Medicine, Pharmacy, Dentistry and Psychotherapy (IMPP). It was developed by an interprofessional working group comprised of members from the German-speaking countries (Germany, Austria, Switzerland) and can be applied to all types of applications and formats in human simulation, at all levels of education and training in the health professions. It is even possible to use this template in contexts above and beyond health (e.g., social work, teacher training), though this was not explicitly the aim.

The template itself has a modular structure. This refers to the chapters listed below and to the individual elements found within them. **In a role script based on this template, the case authors should only select and fill out those sections that are relevant to the implementation or simulation of the scripted case scenario in teaching or assessments. In general, the role scripts should be kept short and pragmatic; everything else that is unrelated or unimportant should be left out.** Explicitly connected with this template is the emphatic request that, when developing role scripts, authors and reviewers critically ask themselves what is truly relevant to a case and should be written down. An exception to this is the specification sheet which must be filled out and placed before the role script itself. This is meant to facilitate categorizations, interchangeability across different institutions, and continual revision.

This template represents the current consensus in the interprofessional working group and is based on relevant publications (see references listed in the paper). It is meant to be as comprehensive as possible, but potentially does not contain all of the elements to exhaustively cover every conceivable instance of a simulated participant in healthcare. Conversely, it is likely that, as mentioned above, not every teaching activity will require every part of this template. For this reason, the template will need to undergo continual development and be adapted to the existing and changing conditions in the separate disciplines and professions. Hence, the template has been licensed under CC BY 4.0 (https://creativecommons.org/licenses/by/4.0/deed.de, accessed on: 13/11/2025) and may be used, in part or in full, by others in their own work as it is, developed further, modified, and disseminated, with proper citation of the original source.

# 1. Specification Sheet

| | |
|---|---|
| Discipline | E.g.:<br>▪ Occupational therapy<br>▪ Midwifery<br>▪ Speech therapy<br>▪ Medicine<br>▪ Nursing science<br>▪ Physiotherapy<br>▪ Psychology<br>▪ Psychotherapy<br>▪ Emergency response services<br>▪ Veterinary medicine<br>▪ Dental medicine |
| Sub-discipline | E.g., geriatric care, pediatrics |
| Occasion / Diagnosis / Situation | E.g., Breaking bad news to a family member in an emergency rescue setting; appointment with a primary care physician for chronic headaches; informational consultation about giving birth at home; initial psychotherapeutic consultation for suspected anxiety disorder; instructions on hygienic hand sanitization |
| Short title in prose | E.g., "Will I be able to dance again?", "I don't want a cesarean!", "My husband is lying unconscious in the garden."<br>(Leave out if no title is needed) |
| Keywords | E.g., wound infection, telephone consultation, shared decision-making, immunization, home birth, interprofessionalism |

| | |
|---|---|
| Educational situation | ▪ Teaching / Assessment<br>▪ Format (e. g. small-group exercise in the Skills Lab, formative OSCE) |
| Degree of difficulty (experienced-based evaluation by the case developer) | ▪ Training/Undergraduate study<br>○ easy<br>○ moderate<br>○ difficult<br>▪ Professional practice (advanced training and postgraduate education)<br>○ easy<br>○ moderate<br>○ difficult<br>Supplemented, where needed, by taxonomic information (e.g., Bloom's taxonomy: analysis, application) |
| Time point in the curriculum | E.g.:<br>▪ Second year of training<br>▪ Ninth semester of undergraduate study<br>▪ 1/3 of undergraduate study completed<br>▪ Postgraduate training in … |

| | |
|---|---|
| Place in the curriculum | E.g.:<br>▪ Module on "Metabolism and Digestion"<br>▪ Elective subject in "Breaking Bad News" |
| Learning objectives / Assessment objectives / Learning outcome / Competency | Formulated according to the common practice in a field, e.g., as a sentence with verb/operator (according to Bloom): "After completing the simulation the students will be able to …." |
| Specifics, if any | E.g.:<br>▪ Designed for several students (e.g., emergency simulation for teams)<br>▪ Designed for use outside of buildings (e.g., mass casualty incident in an emergency rescue setting)<br>▪ Unusual time management (e.g., 45-minute-long teaching simulation in psychosomatics) |

| | |
|---|---|
| Linked with other role scripts / stations | If applicable, any stations or role scripts that come before or after or whose content is directly connected |
| SP casting | ▪ Relevant characteristics (e.g., height, physical build, BMI, hairiness)<br>▪ Inclusion/exclusion criteria (e.g., OP scars, prosthetics, eye glasses)<br>▪ Expertise (e.g., performing the role of specialists)<br>▪ Trigger warnings (e.g., in the case of sexual or violent content) |
| Case developers / Case authors | If applicable, assigned where needed to areas of responsibility, e. g.: 1) Subject matter content, 2) SP program, 3) Didactic approach, 4) Examiners, assessors, etc. |
| Institution | Institution, department |
| Drafted on | xx.xx.xxxx |
| Last revised on | xx.xx.xxxx |
| Last revised by | Surname, first name (institution, date)<br>(An overview of all the changes along with references to the specific chapters is recorded in the Change Log [Chapter 13].) |

## 2. Personal Details regarding the Role's Character

The template begins with the personal details and key information about the character. This entails all of the relevant information based on which the SP trainer develops the role and/or designs the training for the role with the simulated participant. Here, for the sake of clarity, the information is briefly outlined in a table, but is fleshed out further in the third chapter ("Biography, Character and Appearance") where connections are also made (e. g. How living circumstances are impacted by the employment situation). As shown in the example below, the overview in this chapter begins with 1-2 introductory sentences, narrated in the first person, for the purpose of getting into the character, and followed by a table.

Coming after these opening statements is the table summarizing the most important basic categories and going into the corresponding details. The third column provides an option to record comments when specific elements or features of the role should not be changed by casting a certain type of SP or through decisions made by the SP trainers or by introducing diversity into the role, because doing so would distort the medical content, the clinical picture or the teaching/testing objectives. In addition, aspects listed in the content column can be underlined to indicate that they are fixed and should not be altered. Everything that is unmarked or without commentary can be adapted with regard to the entire simulation. A too frequent use of commentary or underlining to rule out changes should be avoided by case developers and addressed, if need be.

***Example: I am Christa Mueller, 53 years old, and I suffer from severe headaches that started about 10 days ago and which do not really go away after taking pain medication. This morning, I went to see my doctor about it.***

| **Category** | **Content** | **Comments** |
|---|---|---|
| First name, surname | Character's full name | |
| Age | ○ Age range<br>(should be medically feasible)<br>○ If required:<br>A date of birth will be chosen by the SP trainer in coordination with the SP<br>**Note:** If medical findings are being shared or the age is relevant to diagnostic or therapeutic procedures, then the date of birth and age must be fixed in advance. An age range would not be sufficient here. | |
| Biological sex | Male, female, other | |
| Gender identity & Sexual orientation | Transgender, queer, heterosexual, homosexual, etc. | |
| Phenotypical appearance & Origin | ○ Skin color, hair color, eye color, etc.<br>○ Continent, country, region, rural or urban | |

| | | |
|---|---|---|
| Personality traits | Using an established model as an orientation is recommended here for reasons of terminology and to minimize over-interpretations and mis-understandings (e. g., the OCEAN model [1, 2, 3], see Attachment) | |
| Migration history | o Voluntary / Flight<br>o Temporary stay abroad | |
| Marital status | Single, partnership (without marriage certificate), married, separated, divorced, partner is deceased | |
| Children | o Number, names and ages of the children<br>o Where the children live<br>o Relationship to the children, e.g., foster children / step-children | |
| Employment situation | o Occupation<br>o Job level, function, location | |
| Educational back-ground / Training | o Level of schooling<br>o University study, vocational training | |
| Free-time activities | o Sports<br>o Hobbies<br>o Volunteer work | |
| Worldview & Religious orientation | Christian (Catholic, Protestant, etc.),<br>Muslim (Shiite, Sunni, etc.),<br>Jewish, Buddhist, atheist,<br>esoteric views, agnostic, etc.<br>o Socialization<br>o Active / passive / membership re-nounced | |
| Living situation | o Information on place of residence/city<br>o Address or living environment | |
| Language | o Foreign languages<br>o Dialects, sociolects | |
| Physical impairments | Wheelchair, prosthetics, eye glasses, hearing aids, etc. | |
| Relationship to rele-vant third parties | Required if any relevant third actors play a health-related role in the simulation (whether by other SPs or in absentia), e.g., sick children or partners, pregnant friends, pets or farm ani-mals in a mono- or interprofessional hand-off of a case, rescue scenarios with spectators.<br>Brief information on:<br>o Relationship (e. g. brother, neighbor)<br>o Attitudes<br>(e.g., "Distanced attitude toward injured dog, latent fears", "Unceasing, over-whelming concern about an only child, protect against all dangers") | |

# 3. Biography, Character and Appearance

In this category, the key information listed above is filled out as concretely as possible so that a brief profile is created to match the case scenario and describe the character role as precisely as possible, along with any artistic license available to the SP.

This section forms the basis upon which the SP identifies with the character and feels their way into the role. The profile accordingly gives the SP important details, for instance, about the character's self-perception, sensibilities and/or social behavior.

In this chapter, case authors support role development and the SP's perception of the role by providing clear answers to the basic questions: who, what, why, where, when and how. For instance, "What are my thoughts about my current living situation?" "What values are important to me?" "What would my friends say about me?" And, "What do I wish for myself in the future in regard to my symptoms?"

The following descriptive details must be written as direct speech in the first person (first person singular) and should be read by the SP and not simply communicated to them by oral means:

1. A brief summary of the key information in Chapter 2
   (not too detailed with all of the information but just a short introduction to the character with only their most relevant aspects)
2. A description of the character's usual outer appearance
   (if applicable, expanding on the information from the previous category in the table, e.g., well-kempt vs. unkempt, hair color and hairstyle, make-up, jewelry and accessories, clothing, gait and posture, items and props [e.g., walking stick, crutches, backpack, purse]), *visible and simulation-relevant aspects of disease are covered in Chapter 4.*
3. Character's conduct during the interaction, e.g.:
   - Reserved due to fear of a bad diagnosis
   - Stubborn and aggressive because the current therapy was unsuccessful
   - Tense and stressed out from the long time spent sitting in the waiting room
   - Afraid due to a relative's need for help (mother, father, child, partner, etc.)
   - Aggressive because the person has realized that something is not right (e.g. on-set of dementia)
4. If relevant: Description of the character's communication style
   (e. g. dialect, simple language, explanations punctuated with many gestures), linguistic symptoms (if needed, with detailed instructions or video links to enable correct presentation) and non-verbal behavior (e.g., gestures, facial expressions, body language, eye contact, proxemics)
5. If relevant: Information on marital status, relationships and/or social situation, domestic conflicts, family or other social conflicts (if needed, give concrete names).

6. If relevant:
    - Traumatic, formative and/or incisive experiences
    - Information about the job situation (e.g., work is primarily done standing or sitting, workload, typical tasks, work/life balance)
    - Interests (e. g. free-time activities, volunteer work)
    - Description of the relationships with third parties relevant to the simulation (played by SPs or in absentia) based on Chapter 2 (e.g., perspective on the health of the other actor, dominant emotions, division of roles, existing duties, expectations, potential for conflicts)

In addition to this, if it is relevant to the role, information can be given about resources or coping strategies, e.g., for dealing with difficult situations or managing with the disease. To the extent possible, the biography should be written in normal everyday language, and the SP should be addressed directly in the first person (e.g., "Luckily, I have never had anything to do with hospitals until now...").

The level of detail in this section is determined by its relevance to the simulation and should not be too excessive. For this reason, constant monitoring should take place to assess which information needs to be set down in writing and to what degree of detail. An overly detailed and rigid biography for the role can hinder the SP or make the character simply impossible to enact.

# 4. Situation and Setting

This chapter describes the reason for the encounter and the current physical and psychological condition of the character during the interaction. In addition, the time and place, including any required equipment, are listed. The relevant information for this is presented in the table.

| | |
|---|---|
| Occasion / Situation | Who should do what to whom, for what reason and where? (repetition of the specification sheet with more details, as needed) |
| Role of the student / examinee | As needed, when a specific role is intended: e.g., long-time primary care physician, supervising midwife, substitute physiotherapist, nursing trainee |
| Place | Localization of the situation<br>(e.g., ambulance, nursing home, preclinical situation at a roadside, therapist's practice, birthing center, psychotherapist's office, hospital, domestic setting [house call, telephone call, telemedical contact], dentist's office, etc.)<br>With the necessary details, such as "rural location a long way from the nearest tertiary medical center." |
| Encounter | Who is in the room? Who is also there? (students, SP)<br>In the case of several people: When does each person enter the scene? |
| Time | Relevant details: e.g., weekday |

| | |
|---|---|
| | If required: time (e.g., night-time emergency) |
| Circumstances | Description of the scenario, e.g.:<br>1. Urgency of the situation<br>2. Initial contact or follow-up (for the latter: Brief description of the relationship to date)<br>3. Referral?<br>4. Crowded waiting room<br>5. Emergency |
| Psychological state | Character's emotional state<br>e.g., relaxed, self-confident, confused, anxious, stressed out, unstable (as for the personal details, using an established model for orientation [e.g., OCEAN model (1, 2, 3)] is also recommended here) |
| Physical state | Character's physical state<br>(e.g., comatose, unresponsive, moribund, sitting, standing, bleeding, limping, in labor)<br>If relevant, with an indication of the trend<br>(e.g., rapidly deteriorating, stable) |
| In the event several SPs are present | If relevant: specific behaviors toward one another (e.g., interruptions, non-verbal aspects like seating positions and looks or glances) |

| | |
|---|---|
| Outfit and accessories of the SP | As with the details in Chapter 3<br>e.g., backpack, formal suit, sun glasses<br>If applicable, a statement about what the SP program will supply and what the SP needs to bring to the simulation. |
| Situation-specific outer appearance | Optical changes due to disease, injury or condition (e.g., make-up, moulages or wax models, fake pregnant belly) |

| | |
|---|---|
| Infrastructure | Necessary equipment/props<br>(e.g., tables, chairs, gurneys, light sources, monitors, cameras)<br>*The general infrastructure is deliberately separated from the specialist equipment and documents in order to make it easier for the various people to fulfill their responsibilities and make sure that everything is in place and working.* |
| Specialist equipment | Information about necessary specialist equipment<br>(e.g., thermometer, mannequin or practice dummy, applicators, EMS vehicle, stethoscope, ECG, pulse monitor, doctor's bag for house calls, lotion, pillows and cushions, dental mirror) |
| Documented findings | Documents and/or handouts for the students<br>(e.g., CTG printout, maternal record of pre-natal and natal care, x-rays, discharge record from rehab, ultrasound, blood work, nursing records, therapy plan, dental status) |
| Configuration of the scene | Placement of chairs, tables, beds, materials, cameras, lights, models, people, etc.<br>Attention needs to be paid here to lighting, visual axes, distances, obstacles, and procedures |

# 5. Prior History

This chapter presents all of the character's backstory that is relevant to the occasion or situation. It is especially important to describe the specifics of the signs and symptoms in as much detail as possible so that a factually precise picture is created, for example, of pain status or symptom severity. This information is prerequisite for defining the SP's latitude during the interaction and describing it in a standardized manner in Chapter 6, Instructions for SP. If the character is neither sick themself nor the reason for the simulated encounter, then the information below is given for the actors who are affected health-wise (e.g., child, father, pet, friend) in such a way that an account worthy of the character can be portrayed by the SP.

Numbers are given in the far-right column to indicate the various degrees of motivation to speak on the part of the character (see Chapter 6.3). This describes the motivation to speak up, not the restricted cognition or ability to communicate due to disease (for this, see Chapters 3 and 4). Alternatively, the different motivations to speak can be color coded.

1 = "Personal motivation to speak" (active, spontaneous reporting)
2 = "Triggered motivation to speak" (no spontaneous, voluntary reporting, but instead willing to provide information if asked)
3 = "No motivation to speak" (the topic's relevance is not apparent, questions are answered in brief, no need is felt to go into greater depth or detail)
4 = "Personal secrets" (shameful topics, no initial reporting and questions are answered only if a safe environment can be created)

| **Domains** | **Specifics** | **Motivation to speak** |
|---|---|---|
| Basic rules | ▪ Anything that is not written in the role script is normal, unknown, or to be negated.<br>▪ Answer only the questions that have been asked. Do not mention any symptoms in the absence of follow-up questions unless explicitly instructed to do so. | |
| Signs and symptoms | ▪ Current complaints, limitations, physical sensations, depressive mood, etc.<br>▪ Pain: localization, intensity and character (if applicable, use a rating scale [VAS, NRS])<br>▪ Concomitant symptoms | |
| Physical characteristics | If fixed values are relevant and if they can be credibly simulated (e.g., by wearing an obesity simulation suit):<br>▪ Height<br>▪ Weight<br>▪ Temperature | |
| Impairments / Restricted participation | Effects on daily life<br>▪ Taking care of one's self<br>▪ Mobility | |

| | | |
|---|---|---|
| | ▪ Social contacts<br>▪ Free-time activities<br>… | |
| History | ▪ Course/cause of the complaints/symptoms/disease<br>▪ If applicable, previous episodes, prior experience with the clinical picture, situation or therapy<br>▪ If applicable, other interactions with the healthcare system<br>(Use relative timelines, e.g., 6 months ago) | |
| Anamneses (depending on professional background and specialization) | E.g.:<br>▪ Pre-existing conditions<br>▪ Risk factors<br>▪ Family history/ family health history<br>▪ Vegetative anamnesis (diet, substance abuse/use [with names/active substances/amounts, if needed])<br>▪ Allergies/Intolerances<br>▪ Vaccination status<br>▪ Lifestyle habits<br>▪ Social history<br>▪ Nursing history<br>▪ Therapy history<br>▪ Obstetric and childbed history | |
| Health literacy | If relevant: Brief description of the character's level of health literacy [4]:<br>1. Find information<br>2. Understand information<br>3. Evaluate information<br>4. Apply information<br>E.g.:<br>▪ Has the person independently researched the current complaints, etc.? (Dimension 1)<br>▪ How nuanced is the person's understanding of health information (including in regard to neighbors, friends, magazines, etc.) and can they apply it to their own case? (Dimensions 2 + 3)<br>▪ Are they able to comprehend the information communicated in the conversation, remember it at home and apply it? (Dimensions 2 + 4) | |

| | | |
|---|---|---|
| Lay person's theory/ subjective concept of disease | Which relevant theories or understanding has the character developed in regard to their body as well as to disease and health:<br>▪ Reason for the disease and/or consultation<br>▪ Correlations and causes between living situation and the illness<br>▪ Risks of the disease<br>▪ Therapeutic success<br>Particularly here, use easily understandable, simple language without technical jargon. | |

# 6. Instructions for SP

The instructions for the simulated participant serve to standardize the start of the conversation and the unfolding of the subsequent interaction. In addition to the desired and/or "permitted" statements, this chapter specifically identifies undesired reactions during the interaction between SPs and students so as not to influence the sequence of events or the students' performance, especially during assessments.

## 6.1 Complementary SP Behavior

Defined here are the general and specific required behaviors (e.g., in the form of learning objectives) which must be enacted by the SP so that the students have the opportunity to attain their own learning objectives, test objectives, learning outcomes, and competencies, (according to the conventions in their field or specialty). The "SP learning objectives" are each meant to complementarily build on the specific requirements placed on the students.

- *Example I (Nursing): The SPs are able to simulate ignorance and/or incomprehension.*
  Complementarily: The students interpret and explain the information to people with health-related limitations and clear needs for nursing care while drawing on basic insights from nursing and reference science.
  (Framework plan of the expert committee as per § 53 PflBG, CE 02: Support of people needing assistance with mobility and daily self-care [Bundesinstitut für Berufsbildung / Federal Institute for Vocation Education and Training, 2020])
- *Example II (Midwifery): The SPs are able to express strong emotions around grief.*
  Complementarily: The graduates support and counsel women and their families in the event of a stillbirth, miscarriage or abortion performed after 12 weeks of pregnancy.
  (Regulations governing the study of and exams in midwifery: Competencies for the qualifying state exam in midwifery [HebStPrV, Annex 1])

- *Example III (Medicine): The SPs are able to actively and of their own accord engage in conflict management.*
  Complementarily: Medical students can perceive and analyze conflicts, specifically address support persons and appropriately contextualize the significance of the underlying sociocultural dynamics and ethical dilemmas.
  (National Catalogue of Competency-based Learning Objectives, Medicine 2.0, VIII.2: Holding medical consultations, learning objective VIII.2-03.1.3 (https://nklm.de/zend/menu (version: 20.12.2024))
- *Example IV (Paramedics): The SPs can credibly enact acute, life-threatening conditions with the help of moulages and make-up.*
  Complementarily: "This means that future paramedics should be qualified to independently apply medical measures to provide emergency care to patients during emergencies and in doing so also apply invasive measure to prevent the deterioration of a patient's condition until medical care can be administered by an emergency care physician or before the administration of other medical care if there is a life-threatening condition or considerable consequences are to be expected. Furthermore, the fully trained paramedics should be able to independently apply medical treatments in the context of collaboration...."
  (Implementation regulations for emergency response training in North Rhine-Westphalia – Part II)

## 6.2 Opening the Situation

Many simulations (and particularly assessments) usually begin with an "opener," a sentence or question following directly after the basic introductions that is meant to kick off the scenario. This should always be written in normal, everyday language and in the form of direct speech (e.g., "I have had an unbelievably bad headache for several days now."). As an opener in preclinical settings, the alarm cue can also be given by the emergency dispatch center.

## 6.3 Conversational Content

For the interaction to unfold as designed, instructional notes or an outline of keywords are needed to train for the role. Differentiation must be made between the various motivations to speak, as already discussed in Chapter 5 regarding the backstory:

1 = "Personal motivation to speak" (active, spontaneous reporting)
2 = "Triggered motivation to speak" (no spontaneous, voluntary reporting, but instead willing to provide information if asked)
3 = "No motivation to speak" (the topic's relevance is not apparent, questions are answered in brief, no need is felt to go into greater depth or detail)
4 = "Personal secrets" (shameful topics, no initial reporting and questions are answered only if a safe environment can be created)

In addition, attitudes toward and opinions on certain (critical) topics can be described here and how the character should react when these are mentioned, for instance:

- Hospital admission
- Psychotherapy
- Addiction and drugs
- Sports and physical exercise
- Giving birth in a hospital vs. outside of a hospital setting
- Immunization
- Need for care
- Stress and strain felt by a family member or other close person
- …

The potential reactions should be described as concretely and true-to-life as possible; if feasible, a spectrum can be laid out identifying different behaviors so that SPs are able to offer a variety of behaviors, especially in the teaching setting. If, over the course of a simulation, the behavior or reactions should change or after a specific period of time something should be stated or expressed, this can be included here also.

### 6.4 Ending the Situation

If the simulation is not terminated by the students or by an external signal (bell, knock on the door, instructors coming in), but rather should be influenced or initiated by the SP, then the corresponding trigger points can be listed here, if applicable, with a specific closing statement in parentheses.

Examples:

- As soon as the diagnosis of XYZ has been given and you have understood it, you express the wish to withdraw ("I would like to have some time alone right now.").
- After mobilization from the bed has been successful and you have taken several steps, you express the wish to rest again for a short while ("I hope this has now been enough for today.").
- As soon as the vaccine has been successfully administered to the dummy's arm, you do not have any further questions and wish to leave the doctor's office. ("That was it for now, right? I need to get back to work.").
- After the CTG has been read and discussed, you wish to end the medical check-up. ("I need to get going now to pick up my child from kindergarten.").

### 6.5 Instructions unrelated to the Role Play

Instructions are given here regarding tasks that are relevant to the organizational procedure, e.g.:

1. Set out a new feedback sheet.
2. Refill materials or return them to their assigned places.
3. Refresh or correct the moulage (e.g., new artificial blood if the wound has been cleaned).
4. Cover up the examination model with a cloth or blanket.
5. Reset the stop watch to zero (if there is one).

# 7. Diagnostic & Therapeutic Interventions

## 7.1 Basic Rules

- The two basic rules from Chapter 5 also apply to the physical examination:
  - Anything that is not written in the role script is normal, unknown, or to be negated.
  - Answer only the questions that have been asked. Do not mention any symptoms in the absence of follow-up questions unless explicitly instructed to do so.
- The common mistakes made by students, which can be expected or that have occurred before, are mentioned in direct connection with each exam or intervention, and the corresponding conduct of the SP in response to it is explained.
- Underwear is never taken off. The intimate privacy of the SP must be protected.
- If something *really* is too painful or the feeling of shame becomes too intense, it is allowed to react accordingly. The commentary comes first from the role of the character; otherwise, it is also possible to come out of character. If applicable, an exit strategy can be arranged in advance (e.g., a specific word/phrase). Dealing with pain and shame should be addressed openly and in detail beforehand, particularly during SP training.
- An SP may ask specific follow-up questions when it is important to explain exactly what is being done and this has been agreed upon beforehand in the context of the simulation. (For example, in the case of a heart auscultation: "Could you tell me exactly what you are listening to and where?")
- Additional detailed information about specific examinations and appropriate reactions (including images and videos) can be appended as needed to the role script or provided via a linked URL. When doing this, SPs should not be overwhelmed with too much technical information or vocabulary.

## 7.2 Diagnostics, Tests & Physical Exams

1) Examination steps
   In the most differentiated manner possible, each examination step is explained in the first column separately and from the individual perspective of the SP as they play the role: What is being done and where?
2) Reactions (non-pathological / "healthy")
   The second column lists the physiological reactions. For examinations of the extremities, the information refers to the healthy side.
3) Reactions (pathological / "sick")
   The pathological reactions are described in the third column. The focus is not on test results (positive/negative), but rather on the behavior and conduct during the test. Non-medical reactions to examination techniques or equipment can also be noted here (asking critical questions, displaying anxiety).

| **Examination steps** | **Reactions (non-pathological / "healthy")** | **Reactions (pathological / "sick")** |
|---|---|---|
| *Abdominal exam (e.g., Appendicitis)* | | |
| Palpation:<br>SP lies outstretched on their back on an examination table. The right side of the lower abdomen is palpated, meaning that the palm of the hand is used to press down on the stomach. | ▪ No pain anywhere in the entire lower abdomen | ▪ Pain on pressure in the lower right side of abdomen.<br>▪ Tense the stomach somewhat in defense (but without any fiercely physical reaction, no rolling up into a ball). |
| Contralateral rebound pain:<br>SP lies outstretched on their back on the examination table. The lower abdomen is pressed with the palm of the hand. When the hand is pulled away, there can be a reaction. | ▪ No pain anywhere in the entire lower abdomen | ▪ Only as the hand releases on the left side is pain felt on the lower right side of the abdomen.<br>▪ Show a slight jerk of the body. |
| Psoas sign:<br>SP lies outstretched on their back on the examination table. The legs are each lifted up separately while the examiner places downward pressure on the leg. | ▪ Both sides tolerate this just fine without any problem. | ▪ Lift left leg up against the pressure without any problem.<br>▪ Lift right leg up against the pressure and distort your face in pain while doing so. |
| *Back Pain*<br>*(e.g., Full picture of a herniated disk on left side, between the 4th and 5th lumbar vertebrae with compression of the L5 nerve root, on left side)* | | |
| Lasègue's sign:<br>SP lies outstretched on their back on the examination table. The leg is passively raised with the knee straight to an angle of 70°. | ▪ Right leg is completely normal.<br>▪ A pseudo-Lasègue or a falsely positive Lasègue's sign is when pain is reported at an angle of 60-70°; this can involve stretching pain in the ischiocrural musculature. | ▪ Passively raised straight left leg → The test is positive when there is stabbing pain in the leg up to an angle of about 45° that shoots from the back into the leg and radiates down to below the knee. |
| Sensory exam:<br>(performed while seated or lying down) The examiner | ▪ Everything feels the same and normal in the right leg. | ▪ The sense of being touched is diminished on the left big toe. |

| | | |
|---|---|---|
| uses their hands to stroke to the right and to the left from top to bottom on the front and back sides and along the sides of the legs. | | (See dermatome reference card)<br>▪ The touch is otherwise perceived equally on both sides. |
| <u>Muscle strength exam (paralysis / key muscle):</u><br>▪ In a standing position: SP raises their toes or lifts their feet up to stand on their heels.<br>▪ Alternative: SP walks on their heels.<br>▪ Alternative: While lying down: SP keeps their knees straight and flexes their feet toward their face. | ▪ Foot can be lifted up onto the heel completely normally on the right side. | ▪ Lifting the foot onto the heel doesn't work on the left side (foot "slaps" when walking).<br>▪ Walking on heels is impossible with the left leg: the left foot doesn't life up.<br>▪ When being examined lying down: Unable to flex the left foot toward the face (simply keep the toes pointing downwards). |
| *Mouth Care Assessment*<br>*(To be filled out within 24 hours of admission for each person in need of care)* | | |
| <u>Items that have been brought along:</u><br>The nurse determines which items have been brought and asks questions about them. | ▪ Tooth brush<br>▪ Tooth paste<br>▪ Upper dental prosthesis, if applicable<br>▪ Lower dental prosthesis, if applicable<br>▪ Prosthetics box with label, if applicable | ▪ You show a well-used tooth brush with bent brushes that you have brought along from home.<br>▪ You state that you have not brought along any tooth paste. |
| <u>Inspection of the lips:</u><br>The nurse examines the lips using a pen light or small flashlight and gives any necessary instructions. | ▪ Pink and moist | ▪ You complain of cracked and dry lips. |
| <u>Inspection of the tongue:</u><br>The nurse asks the SP to open their mouth and examines the tongue using a pen light or a small flashlight.<br>The SP should stick out their tongue. | ▪ Pink and moist | ▪ You report having a dry tongue. |

| | | |
|---|---|---|
| <u>Inspection of the mouth, oral cavity, oral mucosa, teeth, gums:</u><br>The nurse asks the SP to keep their mouth open and examines the entire inside of their mouth using a pen light or a small flash-light. Additional instruc-tions are given, as needed. | ▪ No complaints/pain in the area of the mouth, oral cavity, mucosa, teeth or gums<br>▪ No dryness of the mouth<br>▪ No reduced salivation<br>▪ No halitosis<br>▪ Clean mouth<br>▪ No missing, dam-aged/broken, loose teeth<br>▪ Pink-colored gums | ▪ You report pain while chewing.<br>▪ You have bits of food in the areas between your teeth. Teeth are discolored.<br>▪ When asked, you report having bad breath. |
| <u>Inspection of the cheeks, gums, underside of the tongue:</u><br>The mouth is still open. | ▪ Clean<br>▪ Saliva is present<br>▪ Healthy appearance, nor-mal findings | ▪ You have leftover bits of food in your mouth. |
| <u>Inspection of dental pros-thetics, if applicable:</u><br>The nurse asks that re-placement teeth be re-moved from the mouth for inspection. | ▪ Clean<br>▪ Proper fit | ▪ You state that the dental prosthetic causes pain and doesn't fit properly. You state that you don't regu-larly use the replacement teeth.<br>▪ The dental prosthetic is not clean. |
| <u>Determining the need for assistance with daily oral hygiene:</u><br>The nurse asks questions about oral hygiene habits and practices. | ▪ No nursing assistance is needed for oral hygiene.<br>▪ It is possible to carry out the oral hygiene routine at least 2x per day com-pletely independently at the wash basin.<br>▪ The person is physically and mentally capable of taking care of their oral health independently. | ▪ You state that you are not able to get to the wash ba-sin on your own. |

- Some of the examination steps must be carried out in a specific order of sequence (e.g., abdominal exam). This should only be listed as such when the information is relevant for the SP.
- Some of the examination procedures are very complex. For instance, it can be difficult to describe in words the location of and reaction to pressure points or pain points when assessing movement and should therefore be demonstrated and practiced in detail during role training.
- The examination steps can also be illustrated through images, pain scales, videos or graphics (e.g., dermatome reference cards) so that specific diseases and reaction

patterns can be performed with greater credibility. These can be linked via URL, where applicable.

- In addition to the exams that are expected to occur during the simulation, other tests which are less common, but could be mentioned theoretically if the students bring them up, can also be listed in the table (along with the corresponding reactions).
- It is also possible to include "scripted errors" among the reactions so that SPs conduct themselves ineptly (cover up the part of the body to be examined, take vision test with glasses on) and which the students must correct.
- Perceptions of pain, itching or the like are described colloquially (slight, moderate, severe); quantification as a number is only given in parentheses and if asked for.
- Findings which cannot be simulated are communicated via information sheet, card or by the instructors.

## 7.3 Therapies, Emergencies & Non-diagnostic Interventions

Not all situations in which there is physical contact with SPs follow similar patterns and can be accurately divided into "pathological" or "non-pathological," as done in the examples above. The following table can be used as an alternative, particularly for therapeutic, emergency and non-diagnostic interventions.

1) Intervention
   The steps of the intervention or therapy are briefly identified in column 1.
2) Measures
   The second column can list the measures carried out on the SP in more detail.
3) SP reactions
   The SP reactions to each of the measures are described in the third column. As is the case with diagnostics, it is also possible to note non-medical reactions to therapeutic interventions or medical instruments (critical questions, laughter, fear).

| **Intervention** | **Measures** | **SP reactions** |
|---|---|---|
| *Wound Care (e.g., following an appendectomy)* | | |
| <u>Change of dressing and wound treatment:</u> | Your appendix was removed two days ago by means of laparoscopy, which means that you do not have a large abdominal incision, but rather just three little incisions through which the appendix was accessed. These are located on the belly button and lower abdomen. Up until yesterday you had a drain into which fluid from the wound drained. Today the | You feel pain in the area of the wound and you are afraid of having the band aids removed. You express this to the nurse. When the bandages are removed, dig your fingers into the bedding and contort your face in pain. You do not want to see the wound because you can't tolerate seeing blood. You saw that one band aid was soaked with blood and that |

| | | |
|---|---|---|
| | three band aids which were put on after the surgery are being changed. | has already made you feel nauseous.<br><br>If no information is given, your questions during the procedure are:<br>▪ What does my wound look like?<br>▪ Is everything healing properly? Can anything get infected? And how would I know?<br>▪ Will there be any visible scars? |
| <u>Mobilization</u> | Due to the pain, there can be limitations in mobility. Dizziness is also possible after lying down for so long. However, after your operation it is necessary to get you up and moving around as quickly as possible to avoid complications (e.g., blood clots). The nurse encourages you to get up, assisting you when needed and giving tips on ways to position yourself more easily. | You are lying flat on your back and don't trust yourself to move because you are afraid of feeling pain and dizziness. Standing up on your own seems impossible to you. You ask the nurse for help getting up. Once you are up, you stand and walk with a bent posture. You hold your stomach with your hand, you want to counteract pain by doing so. You are happy to receive tips on ways to position yourself with more ease, but do not ask for this help of your own accord. |
| *Emergency Case Management*<br>*(cABCDE scheme to assess a person found with a laceration to the head after a brawl)* | | |
| <u>c:</u> | After receiving blunt force, you need to be checked for critical bleeding. Your head, torso, extremities and pelvis are externally checked for bleeding. You also need first aid for your head wound. | ▪ You have an open wound on your head.<br>▪ You confirm that you are not bleeding anywhere else.<br>▪ You accept the compression bandage on your head to cover the wound. This pressure bandage can be put on at different |

| | | |
|---|---|---|
| | | time points during the simulation; this is decided by the person making the assessment. |
| <u>A:</u> | Your airways need to be checked to make sure that they are free. Injury to your airways and cervical spine must be ruled out. | ▪ You open your mouth to let it be inspected. Your airways are clear, you are able to breath, cough and speak normally on your own. You have not swallowed anything.<br>▪ You are able to sit up slowly to be treated, with help if needed. You contort your face in pain as you do so; sitting up takes longer than usual. |
| <u>B:</u> | Your breathing is checked. A stethoscope is placed on your upper chest and back, if necessary, to listen to your lungs. The clothing on your upper body is removed or loosened to enable this. | ▪ You state that it subjectively feels harder to breathe and that your breathing is somewhat faster than normal due to the excitement.<br>▪ You accept an oxygen mask if it is offered to you. Your breathing and level of excitement calm down with the oxygen mask. You feel safe and well cared for. |
| <u>C:</u> | Your circulation is checked and any other indications of bleeding are looked for. Blood pressure and pulse are measured. | ▪ Your blood pressure and pulse are normal.<br>▪ The world started to go black on you for a moment, but now you are fully conscious again.<br>▪ You do not feel dizzy. |
| <u>D:</u> | You must be checked to rule out problems with your central nervous system. A basic neurological assessment is conducted using the FAST test (Face, Arm, Speech, Time):<br>▪ Ask for a smile | ▪ You follow all of the directions as the situation demands.<br>▪ You smile when asked to smile.<br>▪ You are able to reach forward with your arms |

| | | |
|---|---|---|
| | ▪ Ask for raised arms<br>▪ Ask for a phrase to be repeated<br>▪ Appropriate response if at least one test is positive | while closing your eyes and rotate your palms to face upwards.<br>▪ You are able to repeat the phrase without any errors. |
| <u>E:</u> | The overall situation is assessed. You are asked about the following information:<br>▪ What happened?<br>▪ When did it happen?<br>▪ How did it come about?<br>▪ What symptoms do you have?<br>▪ Do you have any allergies?<br>▪ Do you take medications or drugs?<br>▪ Do you have any pre-existing conditions?<br>▪ When did you last eat food?<br>▪ Are you in pain?<br><br>A complete physical exam is needed. You must be taken by ambulance to the hospital for further monitoring because a severe traumatic brain injury is suspected. | ▪ These questions do not need to be asked in chronological order; it depends on the person making the assessment. You answer all of the questions with the information from the template.<br>▪ You have a stabbing pain in your head.<br>▪ You refuse to have all of your clothes removed for a physical exam at the scene of the crisis. But you would consent to such a physical exam if you were in a hospital.<br>▪ You agree to be moved to a hospital for further examination. |

## 7.4 Additional Information

| | |
|---|---|
| Instructions for when students make unintended mistakes | e.g., contort your face in pain in reaction to inept or improper movements. |
| Handling moulages | The type and appearance of any moulages – if they are used in the simulation – will have been described above. Include here only the aspects that are relevant to the examinations. |
| Hybrid simulations (Combination of SP with models) | ▪ Go on "standby" but react if you are spoken to.<br>▪ Practice, as needed, how to handle the hybrid segments in advance (including child dummies, mannequins or practice dummies, simulators).<br>▪ Practice, as needed, how to deal with exam materials. (e.g., eye charts) |

| | |
|---|---|
| | ▪ If models are used, clarify at which point pain or pressure, which is not felt in reality, should be mentioned.<br>▪ Setting the values for vital signs on models or examination equipment. |
| Contact with the "outer world" / "Breaking the 4th wall" | ▪ Can information that can't be simulated be asked for from off-stage? (e.g., APGAR test in the case of newborn simulators)<br>▪ Can information be communicated from off-stage during the simulation? If yes, then how?<br>(e.g., written form [papers], verbally ["voice of God"]) |

# 8. Inner Monologue

The inner monologue is a particular form of internal soliloquy and describes what is going through the simulated participant's head. This technique helps the SP focus on getting into the role, take on the mood of the character and envision the imminent situation. The monologue should therefore concentrate on the station's teaching and testing objectives, but may also go beyond them. The inner monologue does not have to deal directly with the disease or the event, rather can deviate instead depending on the circumstances. The inner monologue helps with standardization and is an essential part of training for the role. The inner monologue is written in the first person and may contain sentences that can be chanted internally like a "mantra."

Example: *I have absolutely no clue where the chest pain is coming from.... I never had anything before.... Although dad's second heart attack also just came out of nowhere. Now I don't feel anything anymore — Everything is back to normal again. Not that the doctor will think, I'm pretending. I've been noticing this for several weeks now: it keeps coming back again and again, but it doesn't follow any sort of pattern, even though I have been paying really close attention to it. I will explain it in a lot of detail, then the doctor will see that there is really something going on with me and it is urgent and I am not just rambling on. Hopefully the doctor will have time and won't have to rush off to the next thing. This waiting room is pretty full already. But I want to have this cleared up, once and for all, right now so that I can have some peace of mind again.*

Examples of mantras:

- "I don't want a cesarean!"
- "I really must get back to work."
- "I just can't take it anymore...."
- "I will protect my child regardless of what happens!"

# 9. Diversity in the SP Pool

The following information applies to the SPs themselves and not to the role that is played. In the context of diverse SP pools, the following groups of SPs can have special needs:

- Elderly people [5]
- Disabled people [6]
- Chronically ill people
- Children (ages 5 to 12) [7, 8, 9]
- Adolescents (ages 13 to 18) [7, 8, 9]
- Pregnant women
- People with migration backgrounds
- People with communication difficulties
  (result of disease or disability)

For these special groups of SPs, more time will need to be planned for preparation prior to the simulation. Likewise, extra people to accompany these SPs during and after simulations may need to be taken into consideration.

Basic information for dealing with special groups of SPs:

- Helpful are transparent, clear and generalized procedures for the role play, to the extent possible without spontaneous changes or surprises.
- Overloading the role script should be avoided (despite detailed instructions). Ideally, the simulation will consist of several small informational segments which can be interchanged in order to avoid excessive demands.
- If applicable, appropriate training methods or techniques for memorization (mnemonics) can be given in the script for specific topics.
- When performing simulations, there should be the option of an exit strategy. A gesture, trigger word or short statement can be agreed upon in advance with the SP program or the instructors to signal a stop to the simulation (e.g., "I need a break").
- Pre-defined, universal sentences in the template can be memorized by the SP and used in case of a mental blackout during the simulation ("That is such a long time ago" or "I just can't concentrate right now"). The instructors or examiners who are present then know to intervene and assist. These pre-defined sentences can also indicate to any role-play characters accompanying the SP that they need to take a more active part in the conversation.
- Information or short explanations can be included in the role scripts if there is a risk of students imparting wrong or misleading information to the SPs who are personally affected and cannot evaluate its accuracy.
- Noting the maximum amount of work for the affected group can help in planning and scheduling the actual implementation and performance of the simulations in the teaching and testing settings.
- The template should ideally be barrier-free in its accessibility for SPs (e.g., large font, strong black and white contrast, easily accessible file formats).

Elderly people:

- Adjustments to room setup (see Chapter 4): good lighting in the room, elimination of tripping hazards (electrical cords on the floor, etc.).

Disabled people:

- Attention should be paid to adjusting room setups (Chapter 4), for example, a barrier-free route or elimination of tripping hazards (electrical cords on the floor, etc.).
- If the conversation with the SP takes place with the participation of a third person (e.g., a care giver), a helpful prompt could potentially be needed in the template for this third person, e.g.: "She can speak for herself. Please talk to her directly." This way the focus of the conversation can be turned back to the SP.

Chronically ill people:

- A list of unwanted or disruptive diagnostic findings, which can be found in connection with the chronic disease suffered by the SP (e.g., scars), can assist the SP trainers in casting SPs and during training for the role.
- Depending on the case scenario and the general circumstances of the simulation, an SP's own experience can be helpful to the simulations and feedback and used as a resource or they can be disruptive. Relevant experiences and requirements should be noted.

Children/Adolescents:

- The adult accompanying the SP receives instructions that give them the option to intervene to correct a situation if something "wrong" is done (e.g., "Oh, you mentioned before, that ..."). In the event of such situations (e.g., taking speech therapy case histories and giving advice for children, consultations with pediatricians) the use of accompanying adults is not just methodologically helpful, but also necessary for a credible simulation based on reality.

Pregnant women:

- It is important to ensure that the simulation does not pose too great of a strain on the mother-to-be or the unborn child.

People with migration backgrounds:

- The informational materials and the templates for SPs with language barriers due to immigration must be written in an appropriate and easily accessible style for readers. Other types of media can be used here (images, models, graphics, pain scales with smiley faces). Simplified language or translations of the materials are also possible strategies.
- The use of interpreters is also conceivable and should be noted accordingly.
- The cultural or religious particularities of a role should be highlighted and spoken about with the SP to aim for a credible simulation, to avoid stereotypes and to respect any potential sensibilities that the SP might have.

People with communication difficulties (due to illness or disability):

- Simplified language and multi-modal materials, for instance, with graphics, should be used in these cases.
- Notes referring to family members and the possibility of involving them are relevant here.

# 10. Information for Teaching and Assessment

| | |
|---|---|
| Learning objectives / Testing objectives / Learning outcome / Competency (analogous to Chapter 1: Specification Sheet) | Formulated according to the field's conventions, e.g., as a sentence with verb/operator (according to Bloom): "The students are able to convey bad news in a conversational model specific to a given situation."<br>For high-stakes assessments, the expectations should be set based on relevant academic literature. For instance, specific protocols, guidelines or algorithms can be referred to here. |
| Information and task for students / examinees (if applicable, with an indication of how and when this is communicated) | E.g.: "You are the physician on duty in a hospital emergency room. Three hours ago, an emergency physician handed over a 30-year-old motorcycle rider to you. In the trauma room, in addition to a traumatic brain injury and multiple broken bones, internal hemorrhaging in the abdomen was discovered on sonographic examination. The female patient immediately underwent laparoscopic surgery. The bleeding could not be stopped during surgery. The patient died in the operating room. Your colleague, who just went off duty, called the partner in to the hospital to be informed. He is sitting in the waiting room ready to speak with you."<br>▪ This is sent out via email on the day before.<br>▪ This is posted on the door to the room where the assessment will take place. |
| Organizational information | ▪ Assignment to subject area<br>▪ Assignment in the curriculum (module, lecture)<br>▪ Responsible instructor |
| Instructions for other personnel | If applicable, instructions for others (e.g., helpers, technicians, paramedics) with clear reference to the simulation |
| Time management | ▪ Length of the simulation and any wrap-up discussion<br>▪ Transition phases |
| Feedback & Debriefing | ▪ Feedback or debriefing: yes/no<br>▪ Feedback/debriefing model (e.g., PEARLS, with references to literature, as needed)<br>▪ Specific focus: e.g., shared-decision making, non-verbale behavior<br>▪ Special aspects: e.g., changes in the order of sequence, not much time |

| | |
|---|---|
| | (No description or structuring of feedback and debriefing models, just their identification or information about relevance and about the special aspects when carrying it out.) |
| Information on standardization | E.g.:<br>▪ For equal treatment at stations during assessments, the amount of SP participation in a conversation and their "acting involvement" need to be as uniform as possible to avoid unequal conditions.<br>▪ The simulation of not knowing diminishes in quality over the course of an assessment. Train for this specifically and change out the SP if required. |
| Information on adjustments and scaling | Ideally, based on experience:<br>▪ Possibility to make modifications to fit the degree of complexity or level of difficulty<br>(raise or lower): e.g., by changing the familiar context and occupation, through more exotic, subjective concepts of disease<br>▪ Notes on modifying the station in terms of variety in teaching and testing: e.g., through different psychological stresses, the presence of family members, different previous experiences. |
| Common reactions of students / Inappropriate conduct | E.g.:<br>▪ Although the students' assigned task is only to inform about taking another blood sample, many students expect that the equipment to do so will actually be present and are frustrated when it is not.<br>▪ The students must mediate between two parents on the topic of immunization. Some students join the conflict and sometimes even choose a side, whereby the character and aim of the simulation can change. |
| Experiences based on previous rounds and CAVES | E.g.:<br>▪ Due to the intensity of the exams, changing out the SPs every 4 rounds is recommended.<br>▪ In a case involving cultural competence, students often intermix the views of the role-play character with those of the SPs. The SPs must be protected in these cases, and the simulation stopped in a clear manner with a transition to the feedback session.<br>▪ The role addresses the topic of bullying. It may be necessary to clarify in advance whether the students have had their own experiences with this so that they can prepare themselves or excuse themselves from the simulation to protect themselves from undue stress. |
| Assessment criteria | Statistical analyses of the test scores can be provided for cases that have already been used in assessments (especially in assessments with major consequences for the examinees [high-stakes]) e.g., reliability, discriminatory power, difficulty. |
| Quality assurance | E.g.: |

| | |
|---|---|
| (Tools and results) | MaSP (The Maastricht Assessment of Simulated Patients) [10], NESP (Nijmegen Evaluation of the Simulated Patient [11], Fair_OSCE (Focused Assessment of Interactive Role-play in Objective Structured Clinical Examinations) [12] |

# 11. Information for SP Trainers

Relevant aspects connected to the role can be listed here for the SP trainers, but which may not be necessary for the SPs to know, e.g.:

- Suitable SP training methods for specific types of desired behaviors, with a focus on the competencies and/or learning objectives relevant to the students.
- Typical evolutions in the enactment of the character as a result of the SP's increased experience inhabiting the role.
- If necessary: theoretical contexts for the learning objectives, bibliographical references, medical information.
- Notes on using the equipment (e.g., how do I handle models in hybrid simulations).
- Handouts/materials for SPs about preparation beforehand or wrap-up afterward and notes about usage.

# 12. High-stakes Assessments / State Exams

Cased-based instructions for SP trainers and SPs regarding high-stakes assessments (e.g., state exams) are laid down by the legally responsible testing institutions (Germany: Institut für Medizinische und Pharmazeutische Prüfungsfragen [IMPP]; Switzerland: Institut für Medizinische Lehre [IML]; Austria: Zentrum für Medizinische Lehre [JKU]). These instructions are of legal relevance and cannot be (substantially) altered on site. Local and/or regional assessment standards for a specific profession (e.g., for a medical school's own OSCE) can be included here.

# 13. Change Log

It is important for the further development and quality assurance of role scripts, particularly in the case of assessments (and especially those with high-stakes), to make any changes to the template comprehensible and to clearly record in writing who is responsible ("Who changed what and when?). The following table can be used for this purpose:

| Person | Date | Changes (with indication of chapter or section) |
|---|---|---|
| | | |

# 14. References


1. Gerlitz JY, Schupp J. [Internet]. 2005 [cited 2024 Dec 1]. Research Notes zur Erhebung der Big-Five-basierten Persönlichkeitsmerkmale im SOEP. Deutsches Institut für Wirtschaftsforschung. Verfügbar unter: https://www.diw.de/documents/publicationen/73/43490/rn4.pdf

2. Stemmler G, Hagemann D, Amelang M, Spinath FM. Differentielle Psychologie und Persönlichkeitsforschung. 8., überarbeitete Auflage. Stuttgart: Kohlhammer; 2016. (Hasselhorn M, Kunde W, Schneider S, Reihenherausgeber. Kohlhammer Standards Psychologie).

3. Ziegler M, Horstmann KT, Ziegler J. Personality in situations: Going beyond the OCEAN and introducing the Situation Five. Psychol Assess. 2019 Apr;31(4):567-580. DOI: 10.1037/pas0000654

4. Sørensen K, Van Den Broucke S, Fullam J, Doyle G, Pelikan J, Slonska Z, u. a. Health literacy and public health: A systematic review and integration of definitions and models. BMC Public Health. 2012 Jan;12(1):80. DOI: 10.1186/1471-2458-12-80

5. Smith CM, Sokoloff LG, Alsaba N. Collaborative framework for working with older simulated participants (SP). BMJ Simul Technol Enhanc Learn. 2021 Mär;7(2):112-115. DOI: 10.1136/bmjstel-2020-000613.

6. Thomas B, Courtenay K, Hassiotis A, Strydom A, Rantell K. Standardised patients with intellectual disabilities in training tomorrow's doctors. Psychiatr Bull. 2014 Jun;38(3):132-136. DOI: 10.1192/pb.bp.113.043547

7. Gamble A, Nestel D, Bearman M. Children and young people as simulated patients: recommendations for safe engagement. IJoHS. 2022,1(4). DOI: 10.54531/erqf8206

8. Gamble A, Bearman M, Nestel D. A systematic review: Children & Adolescents as simulated patients in health professional education. Adv Simul. Januar 2016;1(1):1.

9. Kurpat R, Schrewe FB, Sensmeier J, Marschall B. Kinder als SPs. In: Peters T, Thrien C, Hrsg. Simulationspatienten Handbuch für die Aus- und Weiterbildung in medizinischen und Gesundheitsberufen. Bern: Hogrefe; 2018. p. 231-238.

10. Wind LA, van Dalen J, Muijtjens AMM, Rethans JJ. Assessing simulated patients in an educational setting: the MaSP (Maastricht Assessment of Simulated Patients). Med Educ. 2004 Jan;38(1):39-44. DOI: 10.1111/j.1365-2923.2004.01686.x

11. Bouter S, van Weel-Baumgarten E, Bolhuis S. Construction and Validation of the Nijmegen Evaluation of the Simulated Patient (NESP): Assessing Simulated Patients' Ability to Role-Play and Provide Feedback to Students. Acad Med. 2013 Feb;88(2):253-259. DOI: 10.1097/ACM.0b013e31827c0856

12. Brem B, Richter CS, Schnabel K. FAIR_OSCE – Feedback structure for assessment of interactive roleplay in Objective Structured Clinical Exams (Unveröffentlicht). Presented at: 4th Swiss Conference on Standardized Patients and Simulation in Health Care; 2014 Sep 10-12; Bern, Switzerland.

*Attachment 1: The Big Five Personality Traits*

| Abbreviation | Dimension (English) | Dimension (German) |
|---|---|---|
| O | Openness | Offenheit |
| C | Conscientiousness | Gewissenhaftigkeit |
| E | Extraversion | Extraversion |
| A | Agreeableness | Verträglichkeit |
| N | Neuroticism | Neurotizismus |

Table 1. The five main dimensions of human personality according to the OCEAN model. [1]

*Attachment 2: Personality Traits and Their Expression*

| Dimension | Expression of Trait | |
|---|---|---|
| | **Very pronounced** | **Not pronounced** |
| Openness | • multi-faceted<br>• curious<br>• philosophical<br>• creative<br>• more unconventional<br>• reflective<br>• imaginative | • traditional<br>• protective<br>• not very open to new things<br>• insular<br>• conservative<br>• more conventional |
| Conscientiousness | • orderly<br>• deliberate<br>• industrious<br>• punctual<br>• responsible<br>• meticulous<br>• diligent<br>• dependable | • careless<br>• spontaneous<br>• more weak-willed<br>• unheedful<br>• inexact<br>• inattentive |
| Extraversion | • communicative<br>• sociable<br>• dominant<br>• hands-on<br>• spontaneous<br>• genial<br>• cheerful<br>• optimistic | • reserved<br>• calm<br>• likes to do things alone<br>• serious<br>• independent<br>• inhibited<br>• introverted |
| Agreeableness | • in need of harmony<br>• peaceful<br>• cooperative<br>• compassionate<br>• nice<br>• helpful<br>• compliant | • egocentric<br>• obstinate<br>• aggressive<br>• mistrustful<br>• firm<br>• very skeptical<br>• competitive |
| Neuroticism | • stressed<br>• anxious<br>• full of cares<br>• fraught<br>• insecure<br>• self-conscious<br>• pessimistic<br>• sad | • relaxed<br>• content<br>• even-tempered<br>• self-confident<br>• unconstrained<br>• stress-resistant |

Table 2. Personality traits and their expression according to the OCEAN model. [2, 3]
