# Supplementary material for: Development of a standardized role script template for simulated participant scenarios – results of a multi-step consensus process in the German-speaking countries
Source: GMS J Med Educ. 2026 Feb 17;43(2):Doc18. doi: 10.3205/zma001812 (PMC12936913; doi:10.3205/zma001812)
Supplement: Role script template for Simulated Participants (SPs). Example for an assessment in undergraduate medicine [file JME-43-18-s-002.pdf]

Attachment 2: Role script template for Simulated Participants (SPs). Example for an assessment in undergraduate medicine

Attachment 2 to Peters T, Bauer D, Fritz AH, Hahn S, Hempel L, Reck L, Reicherts M, Schönbauer A, Strohmer R, Thrien C, Weber M, Zimmermann A, Pippel E. *Development of a standardized role script template for simulated participant scenarios – Results of a multi-step consensus process in the German-speaking countries.* GMS J Med Educ. 2026;43(2):Doc18. DOI: 10.3205/zma001812

Role script template

for Simulated Participants (SPs)

Example for an Assessment in Undergraduate Medicine

1. Specification Sheet

Discipline	▪ Medicine
Sub-discipline	▪ Surgery (Neurosurgery)
Occasion / Diagnosis / Situation	Referral to a surgical emergency department for weakness and numbness in the arm
Keywords	Cervicobrachalgia, examination of the cervical spine, disk herniation

Educational situation	▪ OSCE
Degree of difficulty	▪ Undergraduate study<br>o Difficult
Time point in the curriculum	▪ 8th semester
Place in the curriculum	▪ Internship, OSCE in Surgery
Learning objectives / Assessment objectives / Learning outcome / Competency	After completing the simulation, the students are able to:<br>▪ Hold a structured and focused conversation to take a case history in the surgical emergency department.<br>▪ Perform targeted neurological exams (checking reflexes, sensorimotor function).<br>▪ Arrive at a suspected diagnosis based on the gathered and observed signs and symptoms.

SP casting	▪ Inclusion criteria:<br>- Sex: female<br>- Character's age: 40-50<br>- Willingness to undergo physical examination<br>▪ Exclusion criteria:<br>- No back or neck problems<br>- No surgeries for herniated disk/stenosis (surgical scars)<br>- No scars from thyroid surgeries
Case developers / Case authors	Angelika Fritz
Institution	University Hospital Essen, Neurosurgery
Drafted on	24/05/2023
Last revised on	19/12/2024
Last revised by	Angelika Fritz

2. Personal Details regarding the Role's Character

You are Petra Schönfeldt, 45 years old, married with 2 grown children (ages 23 and 21), and you work as a bookseller. On an emergency basis, you came to the surgical emergency department. because your symptoms (severe, motion-restricting neck pain, pain radiating into the right arm and down to the hand with weakness and numbness) have clearly gotten worse over the past two days.

**Category**	**Content**	**Comments**
First name, surname	Petra Schönfeldt	
Age	45 years old	possible age range: 40-50
Biological sex	Female	
Personality traits	O – Curious, open<br>C – Conscientious, orderly, dependable<br>E – Deliberate, reserved<br>A – Gracious, cooperative<br>N – Calm, strained	
Marital status	Married	
Children	○ 2 adult children (aged 23 & 21)	
Employment situation	○ Profession: bookseller<br>○ Employed at a book shop in Essen	
Educational background / Training	○ Secondary school graduate (comprehensive school)<br>○ Professional training (bookseller)	
Free-time activities	○ Swimming<br>○ Hiking with husband in the area<br>○ Reading	
Worldview & Religious orientation	○ Passive (baptized Protestant, but does not go to church)	
Living situation	○ Lives in a rented apartment in Mülheim a. d. Ruhr	
Language	German	
Physical impairments	None previously, now the new neck problems	

3. Biography, Character and Appearance

Not counting the births of my two children and maternity leave, I work as a bookseller in a large book shop in the center of Essen, where I also did my training. I like to read newly published titles and sort the books according to genres. Sometimes, I have to reach up high on the shelves to do this.

The pain has now started to radiate into my right arm, from the inner side of the upper arm and the lower arm all the way to my thumb and index finger. For 2 days now, I have noticed a weakness in my right arm such that I can no longer lift or pick things up. As a result, I am having difficulty when sorting the books at work, especially on the high shelves. I also have a

numbness (diminished sensitivity to touch) in the same area where the pain is. There isn't anything that triggers the symptoms.

I can't remember anything that could have caused this. I haven't fallen down or lifted any heavy things. I also haven't received any (pain) injections lately. I woke up one morning and the pain was there.

I also have no pre-existing conditions or prior surgeries. I am actually healthy. My gynecological check-ups were all normal. My primary care doctor prescribed pain medication, along with something to protect my stomach, and physiotherapy, which made things better for a while. Diagnostic tests have not been done. There are no conspicuous/frequent diseases running in my family, and luckily osteoporosis is not an issue.

<u>Appearance</u>
I am dressed fashionably and am well-groomed. I am right-handed.

<u>Appearance of the role-play character during the interaction</u>

I am reserved, calm but under strain due to the pain. Sometimes, as a result of a careless movement of my head (particularly when turning to the right side), there is an electrifying pain in my neck and down my right arm, which makes me pause to catch my breath and for a moment I can't speak.

<u>Employment situation and its effects on daily life</u>

I am a little worried because my ability to move is so restricted. I can no longer perform all the tasks at home or at work, especially any work that has to be done overhead. Combing my hair this morning was also really difficult. I can still basically take care of myself; of course, my husband helps me out, too. But it all takes so long and is so hard. At work I feel myself to be significantly restricted and can only just barely work the high shelves on my own and have to ask for help a lot more often, which is somehow unpleasant for me. I am not driving at the moment because I am scared that a wave of pain will come or that I won't be able to react the way I need to. Fortunately, my co-worker Simone drives me to and from work, and at the moment, she is actually my only social contact outside of my family. I used to like to go swimming before, but that's not possible right now. Actually, the only pleasure that remains is reading on the sofa in the evenings.

4. Situation and Setting

Occasion / Situation	The primary care physician sends Mrs. Schönfeldt to the emergency surgical department due to new additional symptoms and the severe pain.
Role of the student / examinee	Final-year medical student in the surgery department
Place	Emergency outpatient clinic for surgery / neurosurgery
Encounter	Patient waits in the examination room. Final-year student enters the room from outside.

Time	Normal weekday, during the day
Circumstances	1. Urgency of the situation: emergency presentation of patient<br>2. Initial contact<br>3. Referral by primary care physician
Psychological state	Pain-ridden
Physical state	"Stiff" upper body posture due to pain, does not move head

Outfit and accessories of the SP	Fashionable and well-groomed. A thin blouse that can be taken off easily for examination. Underneath it is a brassiere that is not taken off.
Situation-specific outer appearance	Wearing a neck scarf (for warmth)

Infrastructure	Required equipment: examination table with rolling stool, gloves, hand disinfectant
Medical equipment	Reflex hammer<br>(dermatome reference card for roll training)
Documented findings	None
Configuration of the scene	1. Examination table with the rolling stool placed in front of it<br>2. Adequate amount of space to perform the examinations

5. Prior History

**Domains**	**Specifics**	**Speaker motivation**[1]
Basic rules	▪ Anything that is not written in the role script is normal, unknown, or to be negated.<br>▪ Answer only the questions that have been asked. Do not mention any symptoms in the absence of follow-up questions unless explicitly instructed to do so.	
Signs and symptoms	▪ Restricted movement in the cervical spine: no turning of the head, no nodding, no bending the head backwards.<br>▪ Intensity of pain is low at rest, pain is very unpleasant today when moving, in which case there is a stabbing	Generally: 2<br><br>When the shooting pain comes (due to a careless movement of your head) briefly

[1] Classified as:
1 = "Personal motivation to speak" (active, spontaneous reporting)
2 = "Triggered motivation to speak" (no spontaneous, voluntary reporting, but instead willing to provide information if asked)
3 = "No motivation to speak" (the topic's relevance is not apparent, questions are answered in brief, no need is felt to go into greater depth or detail)
4 = "Personal secrets" (shameful topics, no initial reporting and questions are answered only if a safe environment can be created)

	pain in the neck (visual analog scale: 7-8, whereby 0 means no pain and 10 the worst conceivable pain possible).<br>▪ Radiating pain in right arm (inner side of the upper arm and lower arm down to the thumb and index finger) and numbness along the path of the pain (corresponds to dermatome reference C6 → See dermatome reference card).<br>▪ Flexing the right arm is weaker than 2 days ago (biceps).<br>▪ No vegetative symptoms: urination, bowel movements, genitals, are all normal and "as always").<br>▪ No involuntary weight loss in recent months.<br>▪ No fever, no nocturnal sweating (no soaking wet pajamas).	pause and distort your face in pain.
Impairments / Restricted participation	Effects on daily life<br>▪ Possible to take care of yourself, slowed down, clearly restricted at work (putting books on shelves).<br>▪ Mobility: not driving at present.<br>▪ Social contacts → carpools with co-worker to get to work.<br>▪ Free-time activities: not going swimming at present.	2
History	▪ The pain started coming on slowly four months ago and has gotten gradually worse.<br>"I don't recall anything; I didn't fall or do any heavy lifting or anything like that."<br>No injections in the prior history. Woke up one morning and the pain was there.<br>Prior treatment by primary care physician:<br>▪ Slight improvement with NSAIDs (pain medication) and physiotherapy.<br>▪ "The pain meds helped me quite well."<br>▪ For 2 days now as described, with the addition of numbness and weakness.<br>If asked in a follow-up question:<br>Only briefly discuss it! The pain is intensified when coughing and straining.	2<br><br><br><br><br><br><br><br><br><br><br><br><br><br><br><br>3

	Intermittent tingling in the same area where the pain is.	
Anamneses	▪ Family history / family health history: unremarkable<br>▪ Medication history:<br>Taking Ibuprofen right now as needed plus a proton-pump inhibitor (PPI)<br>▪ Allergies/intolerances: None<br>▪ Risk factors: None, alcohol: occasionally 1 glass of wine (every 2-3 days), no cigarettes, no other substances<br>▪ Lifestyle habits: Job, housekeeping shared jointly with husband, children have moved out.<br>▪ Social history: goes swimming with friends<br>▪ Therapy history: until now only physiotherapy in addition to the pain medication.	2
Lay person's theory / subjective concept of disease	1. Reason for the disease: No explanation. (Nothing wrong happened when swimming either.)<br>2. Connections and causations between life circumstances and disease: Physiotherapy wasn't enough, too easy?<br>3. Risk of the disease: permanent paralysis in right arm, which would mean extreme difficulties at work.<br>4. Therapeutic success: Quick healing with an OP, immediate freedom from pain.	2

6. Instructions for SP

The motivation to speak is generally "triggered": Nothing gets reported spontaneously (so as not to influence student performance on the exam), but rather only when asked about. Then you are willing to supply information.

6.1 Complementary SP Behavior

- The SP can simulate ignorance or lack of comprehension.
  (Complementarily: The students are supposed to explain a suspected diagnosis based on the symptoms gathered from the anamnesis and the physical examination.)

6.2 Opening the Situation

In assessment settings, the beginning sentences are standardized and therefore must be stated verbatim:

"My doctor sent me here!"

"For months I have had pain in my neck and arm. For two days now I have noticed that I can't lift things or pick them up from the floor."

"When I shelve books, I have an especially hard time reaching the high shelves."

6.3 Conversational Content

In the beginning, the examinees should conduct a structured, focused anamnesis regarding the new symptoms. While this is taking place, information is willingly provided but only if it is asked for ("a triggered motivation to speak"). No difficulties arise during the conversation.

During the targeted physical examination, the instructions given by the student are complied with. If, for instance, no directions are given to remove the neck scarf, then you keep it on.

- **At the end** of the physical examination, students should explain the next steps to be taken. If no information is forthcoming from the student, then it is permissible to ask the following question as a cue:
  "What will happen next?"
- **If the students do not identify a suspected diagnosis**, then it is permissible to utter the following sentence as a cue:
  "After the imaging, you will tell me the results, won't you?"

6.4 Ending the Situation

The simulation will end after 10 minutes at the sound of a gong, and the students will immediately leave the room.

There will be one minute before another acoustic signal is given to start the next round. The students will read the instructions for their assigned task on the door and then immediately enter the room.

6.5 Instructions unrelated to the Role Play

During the pause between rounds, put all of the clothing, blouse and neck scarf, back on.

7. Diagnostic & Therapeutic Interventions

7.1 Basic Rules

- The two basic rules from Chapter 5 also apply to the physical examination:

- Anything that is not written in the role script is normal, unknown, or to be negated.
- Answer only the questions that have been asked. Do not mention any symptoms in the absence of follow-up questions unless explicitly instructed to do so.

- Underwear is never taken off. Intimate privacy must be protected.
- If something *really* is too painful or the feeling of shame becomes too intense, it is allowed to react accordingly. The commentary comes from the role of the character.

7.2 Diagnostics, Tests & Physical Exams

**Examination steps**	**Reactions (non-pathological / "healthy")**	**Reactions (pathological / "sick")**
Neck Pain<br>*(Full picture of a herniated disk on right side, between the 5th and 6th cervical vertebrae)*		
<u>Mobility of the head:</u><br>SP is seated and should turn the head to the right and to the left, bend it forwards and backwards.<br>Active: SP moves their head independently.<br>Passive: The student guides the head.	▪ Unrestricted motion is possible to the left, to the front and to the back.	▪ When the head turns to the right there is severe restriction (due to pain)<br>▪ This range of motion is "blocked"; stop after turning just 10-20°.
<u>Sensory exam:</u><br>(Performed while seated or lying down) The person conducting the exam, strokes both their hands right and left from top to bottom on the front and back sides and along the sides of the arm.	▪ Everything feels the same and normal for the left arm and left hand.	▪ The perception of touch is diminished on the inner side of the upper arm and lower arm all the way to the thumb and index finger (see dermatome reference card C6).<br>▪ Otherwise, the sense of touch is felt to be the same on both sides.
<u>Test for muscle strength (paralysis / key muscle):</u><br>While seated: SP bends both arms at the elbow and is meant to keep the arms bent in resistance to the downward pressure placed by the student (biceps).	▪ Arm bending on the left side functions completely normally.	▪ Arm bending on the right side fails to resist the pressure.<br>▪ The right arm straightens out when pressure is placed on it by the student. (It cannot be kept in a bent position.)
<u>Arm reflexes:</u><br><u>Biceps</u> (BSR)	▪ With the reflex hammer on the inside of the left elbow (in a relaxed	▪ There is no response for the right elbow → Arm stays as

	position): There is a prompt and normal reflexive response (slight bending).	it is (tense the arm somewhat).
<u>Triceps Tendon Reflex</u> (TTR)	▪ With the reflex hammer above the bent elbow on both arms: There is a prompt and normal reflexive response (indication of arm extending).	
<u>Radioperiosteal Reflex</u> (RPR)	▪ With the reflex hammer just above the inner side of the left wrist: There is a prompt and normal reflexive response (indication of the hand bending toward the inside).	▪ On the inner side of the right wrist there is no reflexive response → Hand stays as it is (tense the hand somewhat).

- Dermatome reference for C6: https://flexikon.doccheck.com/de/Dermatom_(Anatomie)

- Arm reflexes: https://www.youtube.com/watch?v=8auJneb9LCM or (in English) https://www.youtube.com/watch?v=9o4_8Ps5Mlw

7.4 Inner Monologue

I have absolutely no idea why my neck pain has gotten worse or why my ability to move my arm has also gotten worse. I have followed all of my doctor's instructions! At the pool, I have stopped swimming the breast stroke and have really held back. I've really only been in the whirlpool...Was that still too much? Did I do something wrong? I hope it goes away fast. I really want to be able to do everything again. At work I constantly have to ask my co-workers to shelve the books that go on the high shelves for me–which is embarrassing for me. And just regular everyday things are getting more and more difficult. I'm almost unable to hold a hair dryer.

Your mantras:

- "I want be able to do things pain free!"
- "I need to be able to do everything at work again."

8. Information for Teaching and Assessment

Learning objectives / Testing objectives / Learning outcome / Competency	After completing the simulation, the students are able to:<br>▪ Hold a structured and focused conversation to take a case history in the surgical emergency department.<br>▪ Perform targeted neurological exams (checking reflexes, sensorimotor function).<br>▪ Arrive at a suspected diagnosis based on the gathered and observed signs and symptoms.
Information and task for examinees	"You are a final-year medical student in the surgical department. In the outpatient clinic, a 45-year-old woman presents herself as an emergency case.<br>▪ Conduct a structured and focused anamnesis with the patient.<br>▪ Test arm reflexes (BSR, TTS, RPR).<br>▪ Check the patient's sensorimotor function.<br>▪ Communicate an initial possible diagnosis to the patient."<br>This task is posted outside on the door of the assessment room and can be read as soon as the signal (gong) is heard.
Organizational information	▪ Assigned to the sub-discipline: Surgery<br>▪ 8th semester, internship assessment<br>▪ Responsible subject area: Neurosurgery
Time management	▪ Station: 10-minute duration<br>▪ One full run: 10 times
Feedback & Debriefing	not applicable (OSCE)
Quality assurance (tool)	NESP (Nijmegen Evaluation of the Simulated Patient)

9. Information for SP Trainers

Two films are available as examples of role training: One error-free version and another with built-in gaps exhibited by the "examinee": OSCE_C_Cervical Spine-Herniated Disk

10. Change Log

Person	Date	Change (with indication of chapter or section)
