# Supplementary material for: Development of a standardized role script template for simulated participant scenarios – results of a multi-step consensus process in the German-speaking countries
Source: GMS J Med Educ. 2026 Feb 17;43(2):Doc18. doi: 10.3205/zma001812 (PMC12936913; doi:10.3205/zma001812)
Supplement: Role script template for Simulated Participants (SPs). Example for teaching in nursing [file JME-43-18-s-003.pdf]

Attachment 3: Role script template for Simulated Participants (SPs). Example for teaching in nursing

Attachment 3 to Peters T, Bauer D, Fritz AH, Hahn S, Hempel L, Reck L, Reicherts M, Schönbauer A, Strohmer R, Thrien C, Weber M, Zimmermann A, Pippel E. *Development of a standardized role script template for simulated participant scenarios – Results of a multi-step consensus process in the German-speaking countries.* GMS J Med Educ. 2026;43(2):Doc18. DOI: 10.3205/zma001812

Role script template

for Simulated Participants (SPs)

Example for Teaching in Nursing

1. Specification Sheet

Discipline	▪ Nursing Science
Sub-discipline	▪ Psychiatric Nursing
Occasion / Diagnosis / Situation	Voluntary admission of a person to the hospital due to acute symptoms of chronic schizophrenia.
Keywords	Schizophrenia, acute signs and symptoms, nursing assessment

Educational situation	▪ Teaching
Degree of difficulty	▪ Training/Undergraduate study<br>○ Moderate
Time point in the curriculum	▪ 2nd-3rd year of study, degree program in nursing (academic degree combined with state certification)
Place in the curriculum	▪ Required assignment in Psychiatry
Learning objectives / Assessment objectives / Learning outcome / Competency	After completing the simulation, the students are able to:<br>▪ Recognize crises and respond in a manner appropriate to the situation.<br>▪ Engage in communication that is patient-centered and respectful.<br>▪ Apply nursing assessment tools to people with mental illness.

SP casting	▪ Inclusion criteria:<br>- Character's age range: 35-45<br>- Experience portraying complex roles<br>- Willing to also embody the role physically<br>- Able to perform highly emotional scenes (desperation, fear, isolation)<br>▪ Exclusion criteria:<br>- Existing mental illness of one's own or in one's close circle (trigger warning)<br>- Rejection of the role
Case developers / Case authors	Author: Elvira Pippel<br>Reviewed by: Katja H. (Nursing expert for Psychosomatics and Psychotherapy)<br>Sophie R. (Actress, SP Trainer)<br>Ferdinand N. (Actor, SP)
Institution	University of Applied Sciences, Department of Nursing
Drafted on	05/2023
Last revised on	18/12/2024
Last revised by	Pippel, Elvira

2. Personal Details regarding the Role's Character

You are Oliver Schäfer, 35-45 years old, and you have a chronic form of schizophrenia. You are admitting yourself to the hospital today because the voices in your head have become too loud. You are afraid that you will cause harm to yourself or others.

Category	Content	Comments
First name, surname	Oliver Schäfer	To be determined with SP
Age	35 – 45 years old	To be determined with SP
Biological sex	Male	
Phenotypical appearance & Origin	Obese, long and/or unkempt hair, heavy beard growth	
Personality traits	O – Not very open to new things<br>C – Organized<br>E – Calm, reserved, introverted<br>A – Cooperative, peaceful<br>N – Fearful, worried	
Marital status	Single, living alone, not in a steady relationship	
Children	None	
Employment situation	No vocational training, unemployable	
Educational background / Training	10th-grade secondary school diploma, no vocational training	
Free-time activities	No regular hobbies	
Living situation	2-room apartment in a high-rise housing block at the edge of a city	
Language	German	

3. Biography, Character and Appearance

<u>Diagnosis</u>

My name is Oliver Schäfer and I am between 35 and 45 years old. My life fell apart when I was 18. One night at the train station... I can still remember how the fury raged in me like a fire out of control. I committed vandalism like I was the master of the universe and I kept screaming, "I am God!" "I can do anything!" and "I get to decide everything!". It was as if I were untouchable, as if I were the player who makes the rules of the game. But then the police came and arrested me without me understanding it or agreeing with any of it. I was brought to a closed psychiatric unit against my will. In my head there was only chaos, rage and hate. The doctors decided that I was sick and medicated me. A judge permitted them to do it. Forced medication they called it. It was supposed to help with the voices in my head and with my delusions of grandeur. Four weeks went by and the voices got quieter. It was as if the fog in

my head slowly lifted. I was moved to an unrestricted ward. It was the first time that it became clear to me that something was wrong and that … that I... wasn't well.

<u>From the beginning up to now</u>

It all started when I was 16. I was out and about with my friends and we were drinking like everyone did back then. "Binge drinking" was completely normal; it was just part of things. It was our way of celebrating life and feeling free. At some point, someone brought some grass and from then onwards I was in on it. Getting high was fun, it put everyone in a good mood and made them seem attractive. I felt great and invincible. The girls suddenly liked me and that's somehow addicting. But over time something changed in me. Reality as I knew it began to get fuzzy. I began to see the world differently – larger, more important, as if I could understand and control everything. But that was a lie. I lost touch with reality and lost myself in my megalomania. Despite everything, I was able somehow to finish the 10th grade and graduate from school. Only just. And then the night at the train station happened and my confinement on the closed psychiatric ward. There came the diagnosis of schizophrenia. All of that is now many years ago. Today I know that I have a chronic illness. I will never be healthy again. Every day I fight to have a life that seems normal, but I know that nothing is the way it once was.

<u>Social Situation</u>

I live alone in my small, 2-room apartment in a housing block at the city's edge. I don't have a steady girlfriend or any children. I never learned a trade and have also never worked. My illness won't allow for that. I have a legal guardian who has been assigned to me. He helps with everything that has to do with my apartment, the government, and my finances. Hobbies? Not really. Sometimes I read, but that's all. I know other people with the same diagnosis. Sometimes we meet by chance and chat a bit. But I actually prefer to keep to myself. It's simpler that way and I never need to explain anything to anyone. I need my set routine and clearly structured days – without it I would be lost. I never get up before 10 am and never go to bed before midnight. That's the best way for me. I have learned to live with my illness. But to speak openly about it? Never. It makes me angry when people claim that we schizophrenics are dumb or have a split personality. Maybe I hear voices but I am still Oliver Schäfer. I understand everything and I want to understand everything!

<u>Appearance and Impairments</u>

Nothing is hurting me and I don't have any physical pain. I also don't feel that I have become someone else. My personality has not changed. But the medications have changed me. I have gained a lot of weight. I move slowly, not only because I have gotten so heavy, but also because of the drugs' side effects. And I have difficulties concentrating. Sometimes I feel like I have been drugged, my whole body is numb and I can't really feel myself anymore. I can still hold a conversation but sometimes it takes a long time before I understand what has been said and can give a response. My appearance? I don't care about that. Hair and beard, I just let them grow. Shower? If it's necessary. I smoke 1-2 packs of cigarettes a day and I don't want to stop. I like to smoke. It's part of my life, it's just one part of it.

Voices in head

The voices ... they say I'm worthless. Or they say it would be better if I were dead. Some voices also talk to each other, and other voices comment on what I do. And then there are those that give me commands: "Jump off the bridge!" or "Take this knife and plunge it into your chest!" Especially bad are the voices that want me to hurt other people. That scares me the most. I have often thought about taking my own life but only because the voices have told me to. Thankfully, nothing has happened up to now. This is also because of my regular hospital stays. There I have learned how to cope better with the voices. They will never go away completely, I know that. But I know of ways to make them quieter. Sometimes it's enough to sniff a little bottle of ammoniac, or cause pain to my wrist, or eat a chili pepper. Yet, every year there is at least one phase when I have myself admitted or am committed by the Psychiatric Institute's outpatient clinic (PIC). It's at those points when I am really scared that I will give in to the voices and hurt myself or other people. It is particularly hard when new voices show up. That causes me to become desperate and then I need help in a hospital.

Medication and Outpatient Help (PIC)

Each month, every 28 days exactly, I go to the Psychiatric Institute's outpatient clinic (PIC) for the injection of my maintenance drug, Abilify Maintena®. Before, I always had to remember to take my medications, but now I get my injection once a month and have more freedom and a better quality of life as a result. I also have medication on hand to take as needed for when it gets really bad. I experimented for a long time until I found the right dosage to help me in the acute phases. Plus I take slow-release Quetiapin tablets before going to bed at night. They dampen my excitement and lessen the tension inside me. My hallucinations and mood also get better as a result.

The PIC takes care of patients like me. All of us require an especially broad and intensive offering of therapy and assistance. Doctors, psychologists, social workers, nurses and occupational therapists all work together at the PIC. The PIC monitors me and I don't need to be in the hospital as often or as long. I get help and support in managing crises and stress, even over the telephone. Every now and then I attend individual or group therapy sessions. I like the memory training. Last year I was even part of the cooking class and went on a hike. I think it’s good that I also receive the support I need to cooperate with my guardian when there are questions or things aren't clear. The therapy at the PIC is accessed with a referral from my doctor and my health insurance covers the costs.

Hospital Stays and Autonomy

There are times when I just get scared. The voices get too loud, and I can't control them anymore. Then I go to the hospital and have myself admitted. Despite this, each time I am in the hospital I am worried about if and when I can go back home again and whether my guardian will agree to it. My greatest fear is that I will lose my autonomy. In the past years I have become an expert in my disease; I know everything about it. I always want to have a say in the decisions about my treatment. No one is allowed to tell me what to do or force me to do anything! I am dependable when it comes to my illness. And I'm satisfied when I receive help and the voices can be controlled. I do everything to make myself better as quickly as possible.

Relationship to Nursing Staff

Over the years I have come to know many nurses. Some I've known since the beginning. I trust them blind. But it took many years to build that trust. I am always skeptical about new people, mistrustful and reserved. I don't know who they are, what they are thinking or if they want to take my freedom away from me. I stay quiet and just watch and wait to see. The other people have to show me first that I can trust them. I don't know if other people take me seriously or understand me, they have to prove they do first. Trust takes a long time for me, and people have to convince me first. But if I feel safe and okay, I like to participate in the hospital activities. I know that I get help in the hospital and that it gets better. That was not always the case, I know that. I attend group activities, the early morning exercises and the talk therapy. I know that these activities help distract me from the voices. And I do everything so that I can go back home! Because I WANT to go home!

4. Situation and Setting

Occasion / Situation	A person voluntarily admitted themself to the hospital due to acute symptoms of chronic schizophrenia.
Role of the student / examinee	Undergraduate nursing student
Place	Hospital: unrestricted psychiatric ward
Encounter	A person with schizophrenia experiencing acute symptoms is in the hospital room. The nursing student enters the room from the outside.
Time	No particular workday, early shift (during the doctor's round)
Circumstances	Urgent, unforeseen situation
Psychological state	High level of psychological stress, tormented, voices seem threatening
Physical state	Seated on the bed, slumped position
Outfit and accessories of the SP	To be provided, as needed, in advance in coordination with the SP:<br>Obesity simulation suit, wig, beard, old und scruffy clothes, pack of cigarettes
Situation-specific outer appearance	Obese, facial hair growth, uncombed hair, unwashed, unkempt finger nails, heavy smoker
Infrastructure	Hospital room:<br>1 bed,<br>1 night table,<br>1 table with 2 chairs
Medical equipment	1x nursing history (checklist, condition at admission)<br>1x extended Barthel Index<br>1x Nurses Global Assessment of Suicide Risk (NGASR)
Documented findings	1x previous extended Barthel Index<br>1x previous case history<br>1x medication schedule

5. Prior History

<table>
<tr><th>Domains</th><th>Specifics</th><th>Speaker motivation[1]</th></tr>
<tr><td>Basic rules</td><td colspan="2">Anything that is not written in the role script is normal, unknown, or to be negated.
Answer only the questions that have been asked. Do not mention any symptoms in the absence of follow-up questions unless explicitly instructed to do so.</td></tr>
<tr><td>Signs and symptoms</td><td>Fear, torment, worry
Psychological stress caused by loud voices in head</td><td rowspan="4">In regard to unfamiliar nursing staff:

Generally: 3

If trust is established: 1

If no trust is established: 2 or 3</td></tr>
<tr><td>History</td><td>The diagnosis of schizophrenia has been made since age 18
Every year there is at least 1 episode where a hospital stay is necessary (2 episodes max.)
Patient is considered to be experienced with psychiatric care.</td></tr>
<tr><td>Anamneses</td><td>Mental illness does not run in the family.
Cause: Smoking cannabis
Occasional alcohol consumption
Heavy tobacco consumption
(1-2 packs / day)</td></tr>
<tr><td>Lay person's theory / subjective concept of disease</td><td>The person knows that the schizophrenia was caused by drug use. He still asks himself after many years of illness and therapy, "Why did I ever start smoking weed back then?"</td></tr>
</table>

6. Instructions for SP

6.1 Complementary SP Behavior

You may simulate dismissive behavior if, from your point of view, no trust-based atmosphere has been created by the students. This is apparent in that you have no motivation to speak of

[1] Classified as:
1 = "Personal motivation to speak" (active, spontaneous reporting)
2 = "Triggered motivation to speak" (no spontaneous, voluntary reporting, but instead willing to provide information if asked)
3 = "No motivation to speak" (the topic's relevance is not apparent, questions are answered in brief, no need is felt to go into greater depth or detail)
4 = "Personal secrets" (shameful topics, no initial reporting and questions are answered only if a safe environment can be created)

your own accord. Also, you respond only briefly in response to questions. You do not share any personal secrets and do not mention any shameful topics.

6.2 Opening the Situation

You do not initiate any conversations on your own. Based on the situation, you react to what the students say to you. You have been in this situation many times before but you are wary of each new person.

6.3 Conversational Content

In your case the students are supposed to assess your current physical and mental state in order to plan your nursing care. To do this, you will be asked questions that you are meant to answer. You do not report anything of your own initiative and your responses to questions are brief or monosyllabic. Depending on how the interaction with the student develops, you respond more openly or remain cautious and distrustful.

**Topic**	**Behavior**
Admission to hospital	You accept this as necessary, but the desire to leave again is, in all events, present.
Psychological stress	Incredibly high at the moment; the voices are unbearable. You are afraid that you will kill yourself or someone else.
Drugs & Addiction	Why did I ever smoke weed?
My illness	I am ill.<br>I am the only one to blame for it.<br>No one forced me to start smoking cannabis; it was my own decision.<br>I am very familiar with my illness. I have dealt with it for many years now.
Care and therapy plan	I know best what's good for me; no one is allowed to make decisions without my input!
Adherence to therapy	I do everything to make myself get better.<br>I have my medication injected so that I can't forget to take it.
Autonomy	Self-determination is the most important thing for me!
Death	I am afraid of death. I don't want to die. I would never voluntarily commit suicide.

6.4 Ending the Situation

The simulation ends after a maximum of 20 minutes. The students are given an acoustic signal and the instructions to wrap the situation up within the next minute.

Note:

If at any point you should feel uncomfortable and wish to end the conversation of your own volition, you may utter the following sentences to extract yourself from the situation and to

turn away from the student: *"It's all just gotten too much for me. I would like a moment to myself alone. Please come back later."*

7. Diagnostic & Therapeutic Interventions

No physical examination is required during this simulation. It only involves spoken questions asked of you.

However, it could happen that students want to try to apply measures anyway, for instance, to measure your blood pressure or feel your pulse. In these cases, you say: *"I don't want that right now. Maybe later."*

8. Inner Monologue

"I can't go on anymore. I need help. I can't do it alone. I don't want anything bad to happen. I just can't get the voices to quiet down. There are too many of them, too many evil voices and thoughts. I don't want to hurt anyone, but I don't know if I can control myself. The voices … They are creeping up on me, they are clawing themselves into my back, they just won't let me go! It's so loud, so damn loud. I don't want it! I want them to be quieter! I will get help in the hospital. I know that."

Your mantras during hospital admission:

- "I want the voices to quiet down."
- "I do not want anything bad to happen."
- "I want to be taken seriously."

9. Information for Teaching and Testing

Learning objectives / Testing objectives / Learning outcome / Competency	Expectations:<br>▪ Establish trust<br>▪ Show empathy<br>▪ Person-centeredness<br>▪ Protection of autonomy<br>▪ Application of communication techniques appropriate to the situation<br>▪ Perform nursing assessment in a psychiatric setting
Information and task for students	"You are a nursing student who was assigned 2.5 weeks ago to an unrestricted psychiatric ward for practical training. 30 minutes ago, Oliver Schäfer admitted himself for treatment and was brought to your ward. Mr. Schäfer is known on your ward and has been coming to the hospital regularly since he was 18 due to his schizophrenia. At every admission, the physical and mental states are assessed. But because the daily physician's round is underway right now, you undertake to carry

	out the nursing assessment. You gather Mr. Schäfer's case history for the purpose of assessing and planning his need for care, record the extended Barthel Index, and assess his present risk of suicide."
Instructions for others	E.g., for a tech crew:<br>Preparation of the audio-video debriefing system<br>If relevant, setting up mobile cameras in the simulation room
Time management	▪ Pre-briefing: around 15 minutes<br>▪ Simulation: around 15 – 20 minutes<br>▪ Debriefing & feedback: about 20 - 25 minutes
Feedback & Debriefing	1. Debriefing by the instructors (3D-Model of debriefing)<br>2. Feedback from SP (WWW.Feedback.DE-Modell), Focus: Building trust<br>3. Feedback from peers (WWW.Feedback.DE-Modell)

10. Instructions for SP Trainers

YouTube channel of an affected person: *Living Well with Schizophrenia*

Material to prepare for the role:

"I'm in psychosis right now": https://www.youtube.com/watch?v=GPcidLBluqM

11. Change Log

Person	Date	Changes (with indication of chapter or section)
Elvira Pippel	18/12/2024	Specification Sheet, Speaker's motivation, Diagnostic and Therapeutic Interventions, Inner Monologue
